# Additive Effect of the Composition of Endophytic Bacteria *Bacillus subtilis* on Systemic Resistance of Wheat against Greenbug Aphid *Schizaphis graminum* Due to Lipopeptides

**DOI:** 10.3390/life13010214

**Published:** 2023-01-11

**Authors:** Sergey D. Rumyantsev, Valentin Y. Alekseev, Antonina V. Sorokan, Guzel F. Burkhanova, Ekaterina A. Cherepanova, Ravil R. Garafutdinov, Igor V. Maksimov, Svetlana V. Veselova

**Affiliations:** Institute of Biochemistry and Genetics, Ufa Federal Research Centre, Russian Academy of Sciences, Prospekt Oktyabrya, 71, 450054 Ufa, Russia

**Keywords:** endophytes, lipopeptides, surfactin, iturin, phloem-feeding insects, aphicidal activity, additive effect, phytohormones, *PR* genes, induced systemic resistance (ISR)

## Abstract

The use of biocontrol agents based on endophytic bacteria against phloem-feeding insects is limited by a lack of knowledge and understanding of the mechanism of action of the endophyte community that makes up the plant microbiome. In this work, the mechanisms of the additive action of endophytic strains *B. subtilis* 26D and *B. subtilis* 11VM on the resistance of bread spring wheat against greenbug aphid *Schizaphis graminum,* was studied. It was shown that *B. subtilis* 26D secreted lipopeptide surfactin and phytohormones cytokinins, and *B. subtilis* 11VM produced iturin and auxins into the cultivation medium. Both strains and their lipopeptide-rich fractions showed direct aphicidal activity against greenbug aphid. For the first time, it was shown that *B. subtilis* 26D and *B. subtilis* 11VM in the same manner, as well as their lipopeptide-rich fractions, activated the expression of salicylate- and ethylene-dependent *PR* genes, and influenced plant redox metabolism, which led to an increase in plant endurance against aphids. The composition of endophytic strains *B. subtilis* 26D + *B. subtilis* 11VM had an additive effect on plant resistance to aphids due to an increase in the number of endophytic bacterial cells, and, as well as due to the synergistic effect of their mixture of lipopeptides − surfactin + iturin, both on the aphid mortality and on the expression of *PR1* and *PR3* genes. All these factors can be the reason for the observed increase in the growth of plants affected by aphids under the influence of *B. subtilis* 26D and *B. subtilis* 11VM, individually and in composition. The study demonstrates the possibility of creating in the future an artificial composition to enhance plant microbiome with endophytic bacteria, which combines growth-promoting and plant immunity stimulating properties against phloem-feeding insects. This direction is one of the most promising approaches to green pesticide discovery in the future.

## 1. Introduction

The greenbug aphid (*Schizaphis graminum* Rondani) is a non-migratory, polyphagous aphid found throughout the world [1,2]. Greenbug aphid causes the greatest damage to winter and spring wheat, winter and spring barley, rye, oats, corn, sorghum, pearl millet and rice, which leads to serious economic losses in agriculture. Currently, this pest is controlled by the use of chemical insecticides. However, this can lead to pest resistance and soil contamination.

An effective way to increase the resistance of grain crops against aphids is the use of biological control agents based on plant growth–promoting bacteria (PGPB), especially endophytic bacteria that are able to mutually live inside plant tissues and form a long-term host defense against pathogens and pests, which is known as priming [3,4,5]. Endophytes, including endophytic bacteria, exist in various organs, tissues, and the intercellular spaces of plants, without causing direct signs of diseases [6]. Endophytes have many advantages over soil microorganisms, since, living in the internal tissues of plants and occupying a special ecological niche, they have a stronger and more lasting effect on the host [6]. Endophytes that colonize plant tissues are considered as naturally occurring biological control agents [6].

Research is currently focused on the complexity of relationships between host plants and their endophytes [7]. However, the mechanisms by which endophytes protect plants from abiotic and biotic stresses remains unclear [6,7,8,9]. Moreover, the main difficulty in studying plant-endophyte interactions is associated with the diversity of the microbial community. It is currently believed that the beneficial properties of bacterial endophytes are realized through direct and indirect defenses mechanisms, due to the secretion of a wide range of different metabolites [4,6,9]. Direct mechanisms of plant protection by endophytes are implemented mainly through the secretion of metabolites with antibiotic activity (mainly antimicrobial peptides (cyclic lipopeptides (LPs), siderophores, polyketides, etc.) and of hydrolytic enzymes—chitinases, glucanases, proteases, lipases, amylases, lactamases, and cellulases, capable of destroying the cells of pathogenic fungi and a number of other compounds. Indirect mechanisms are associated with competition with pathogens for space and nutrients and with the ability of endophytes to stimulate plant growth and induce resistance against diseases and pests, through the secretion of metabolites with growth-regulating activity, for example, phytohormones such as auxins, cytokinins, gibberellins, abscisic (ABA), salicylic (SA) and jasmonic acids (JA), as well as volatile organic compounds and others [4,6,10,11].

Currently, plant immunity is considered as an integral system that includes the action and interaction of a complex holobiome, in which plant and microbes in the phytosphere can prepare the final result when faced with biotic stress [9]. The activation of the immune system of plants is carried out by stimulating the protective forces through induced systemic resistance (ISR) [12,13]. The results of different research groups have provided strong evidence for the role of endophytes in triggering ISR against pathogens and pests, as summarized in recent reviews [6,7,9]. Bacterial endophytes-mediated ISR is regulated by bacterial-produced hormones such as SA, ABA, JA, and ethylene [5,13], as well as cyclic LPs [9,12], and is characterized by the rapid and early accumulation of reactive oxygen species (ROS), the activation of redox-sensitive transcription factors, the upregulation of genes encoding pathogenesis-related proteins (PR proteins), the accumulation of secondary metabolites, and other reactions of this whole metabiome to pathogen/pest attacks [4,5,6]. The triggering of ISR by non-endophytic PGPBs is well studied and occurs via microbe-associated molecular patterns (MAMPs)—metabolites with eliciting activity such as flagellin, lipopolysaccharides, siderophores, etc. [5,6,9,12]. However, the current research on endophyte metabolites in biological control is mainly focused on antibiotic and growth-promoting activity, and the reports on the eliciting role of metabolites of bacterial endophytes are relatively limited [6].

Lipopeptides (LPs) are one of the main groups of bacterial metabolites that are of great interest to scientists due to their multifunctionality and are currently being actively studied. Lipopeptides are synthesized by non-ribosomal peptide synthetases (NRPSs) and consist of a lipid tail linked to a short linear or cyclic oligopeptide [14,15]. *B. subtilis* lipopeptides—surfactins, iturins, and fengycins—have a wide spectrum of biocidal (bactericidal, fungicidal, and insecticidal) effects [14,15]. Lipopeptides exhibit antagonism towards other organisms due to their ability to bind the lipid bilayer of the plasma membrane and change permeability or destroy its structure, forming pores in it, as in the case of fengycin and iturin, or dissolving it, as in case of surfactin [14,15,16,17]. Numerous studies confirm the key role of LPs in the antibiotic activity of bacterial strains, which plays an important role in pest control [9,14,15]. Currently, the insecticidal properties of LPs of bacteria *B. subtilis* are being actively studied and certain success has been achieved. There are studies that show the insecticidal activity of LPs against the orders Diptera, Coleoptera, Hemiptera, and Lepidoptera [11,18,19,20,21]. However, scientists are interested in the elicitor role of these metabolites in triggering protective signaling pathways in plants [10,17,20,22,23].

To date, many works have been accumulated that show the elicitor role of LPs in triggering signaling pathways in various plants against a wide range of pathogens [23,24,25,26,27]. Fengycin and surfactin induced a hypersensitivity reaction and cell death, caused JA/ethylene-, ABA-, and auxin-dependent signaling pathways, which blocked the growth and development of the pathogen at an early stage of pathogenesis [24]. Fengycins induced gene expression of the phenol-propanoid pathway and ethylene signaling pathway in plants [25,26], while surfactins triggered a number of components of the oxylipin signaling system and the salicylate signaling pathway [27]. Recently, several works have appeared on the role of iturin in triggering ISR against pathogens, where this LPs induced ethylene and SA signaling pathways, increased the activity of lipoxygenase and peroxidase in citrus fruit and cherry tomato fruit [23,25,26]. However, information on the elicitor role of LP in triggering ISR in plants against insect pests is limited to one study in which the bacillopeptin-producing *B. velezensis* YC7010 endophyte induced resistance in rice to brown leafhopper (*Nilaparvata lugens* Stål) via triggering SA and FA signaling pathways, deposition lignin and the synthesis of secondary metabolites [22]. However, information about the elicitor role of LPs in triggering ISR in plants against insect pests is limited to one study in which the bacillopeptin producing endophyte *B. velezensis* YC7010, induced resistance in rice against brown planthopper (*Nilaparvata lugens* Stål) via triggering SA and JA signaling pathways, lignin deposition, and the synthesis of secondary metabolites [22]. There is no information that LPs are capable of inducing systemic resistance in plants against aphids.

Thus, much of the success of endophytes is associated with the production of a wide range of metabolites. In addition, biological control agents composed of multiple strains of the same species or of different species of bacteria are known to enhance and broaden their protective spectrum [10,11]. However, it should be noted that studies related to the application of metabolites from composition of endophytes with a different pool of metabolites in strengthening plant resistance are still limited and need to be to be studied and supplemented in future research. The study of the combination of several endophytes can be closer to the real situation in nature, when plants are inhabited by a microbial community. In addition, the question of the regulation of the balance between the promotion of plant growth and the induction of defense mechanisms by compositions of endophytes that have different effects on plants needs requires clarification. Exploring the connection between these two events should be the focus of future research aimed at creating artificial plant microbiomes. [9]. Moreover, studying the additive mechanisms of action of bacterial mixtures will bring us closer to decoding the functioning of the whole plant microbiome. It is assumed that the induction of plant hormonal systems may play a decisive role in this [10,13].

The aim of this work was to study the possible mechanisms of the additive action of endophytic strains *B. subtilis* 26D and *B. subtilis* 11VM on the resistance of bread spring wheat against *S. graminum*, which could be associated with the endophytic properties of bacteria, with the synthesis of various metabolites by bacteria, with growth-promoting and immunostimulating activities of bacteria. The main question of this study was to determine whether LPs can induce systemic resistance in wheat against *S. graminum* and whether LPs in the mixture can enhance this resistance. Using high performance liquid chromatography (HPLC), it was shown that the *B. subtilis* 26D and *B. subtilis* 11VM strains synthesize surfactin and iturin lipopeptides, respectively, into the cultivation medium. Growth-promoting and immunostimulating concentrations for bacterial strains and LPs were assessed individually and in compositions. Endophytic strains *B. subtilis* 26D and *B. subtilis* 11VM had a positive effect on the resistance of wheat plants against greenbug aphid, increased plant tolerance, influenced plant redox-metabolism, and triggered ISR. The composition of strains *B. subtilis* 26D + *B. subtilis* 11VM showed an additive effect in inducing growth and resistance of wheat plants against greenbug aphid. Our results suggest that when compiling bacterial compositions, it is necessary to consider the spectrum of metabolites, which will determine the critical properties of bacteria, such as the number of endophytes in plant tissues, growth-promoting activity, and the ability to trigger various hormonal signaling pathways.

## 2. Materials and Methods

### 2.1. Research Objects

Bacteria: The gram-positive aerobic *B. subtilis* 26D (designated as Bs26D) and *B. subtilis* 11VM (designated as Bs11VM) strains from the collection of the Laboratory of Biochemistry of Plant Immunity of the Institute of Biochemistry and Genetics Ufa Federal Research Center Russian Academy of Sciences (UFRC RAS) (http://ibg.anrb.ru/wp-content/uploads/2019/04/Katalog-endofit.doc, accessed on 10 November 2022) was used. Bacteria were grown on liquid lysogenic broth (LB) medium (1% tryptone, 0.5% yeast extract and 0.5% NaCl) at 28 °C using laboratory shakers (120 rpm) within 72 h until complete sporulation.

Aphids: Insects (*Schizaphis graminum*) were sampled as a population in the summer 2020 from wheat plants that had never been treated by pesticides, in the Ufimsky district of the Republic of Bashkortostan (54°46′00.4″ N 56°00′57.8″ E). The aphid population was massively grown without differentiating individual aphid clones on young seedlings of bread spring wheat (*Triticum aestivum* L.) variety Salavat Yulaev (SY) in isolated pots with sterile soil (heated to 180 °C for 1 h before planting) in KBW E6 plant growth chamber (Binder GmbH, Tuttlingen, Germany) with 16 h light photoperiod at 146 W/m^2^ PAR and 24 °C/20 °C (day/night). 

Plants: The objects of this study were bread spring wheat plants cv. SY, which, according to previous studies, showed moderate susceptibility to *S. graminum* [28]. Seeds were obtained from the Bashkir research Institute of Agriculture—subdivision of the Ufa Federal Research Centre of the Russian Academy of Sciences. In addition, the SY variety is a new breeding variety of the Republic of Bashkortostan, Russia, which is grown in the climatic conditions of the forest-steppe of the Southern Urals, shows high baking qualities and high profitability.

### 2.2. Bioassay of the Endophytic Properties of the Bacillus Strains

The endophyticity of the studied strains was assessed by counting the colony-forming units (CFU) of microorganisms in the sterile SY variety of wheat plants obtained from mature embryos. Wheat seeds were sterilized with 10% hydrogen peroxide solution for 1 min and then germinated for 24 h at 24 °C in sterile Petri dishes to obtain embryos. Then, the embryos were isolated from the endosperm and placed on the 7% agar Murashige–Skoog medium containing 125 U/mL streptomycin and 125 U/mL penicillin, the embryos were grown for 2 days with a 16 h light photoperiod in the KBW E6 plant growth chamber (Binder GmbH, Tuttlingen, Germany). After that, the seedlings were transplanted onto Murashige-Skoog medium without antibiotics and grown in the plant growth chamber for another 8 days. Sterile plants were inoculated with 10 μL of suspensions of Bs26D and Bs11VM strains (10^8^ cells/mL) by applying to the middle part of the leaf. In the variant with the composition of strains Bs26D + Bs11VM, 5 μL of a suspension of each strain was applied to 1 plant. The number of CFU of microorganisms were assessed in 100 mg homogenate of surface-sterilized wheat shoots or roots on the 7th day after the inoculation. Surface sterilization was performed as previously described [29]. Three consecutive 10-fold dilutions of the resultant homogenate were then performed. CFU were counted in the aliquots (30 µL) of second and third dilutions, and their number was recalculated per g of fresh plant weight [29]. In the variant with the composition of strains Bs26D + Bs11VM, colonies that grew in Petri dishes in the third dilution (about 10 colonies per dishes) were analyzed by RAPD-PCR using random primers Lmbd8 5′-GGGCGCTG-3′ for confirmation of the identity of the obtained reinoculants to the original strains. Bacterial DNA from wheat plants was isolated using 1% lysing solution (l% Triton X100, 1% Tween-20, 1% Chelex 100) [30].

### 2.3. Bioassay of the Antagonistic Activity of the Bacillus Strains

The culture of the antagonist strain was streaked on the surface of the agarized LB growth medium in the Petri dish with the 10 μL loop. Plates were incubated for 24 h at 27 °C in Gilson Digital MiniIncubator (Gilson, China). Then, the culture of the test-strain was inoculated perpendicular to the stroke of the grown strain-antagonist. Double cultures were incubated for 24 h at 27 °C, after which the zone of inhibition between perpendicular bacterial lines was measured [31].

### 2.4. Bioassay of the Phytohormone Content in the Liquid Culture Medium of B. subtilis

The liquid culture medium obtained by cultivating Bs26D and Bs11VM was collected at the late logarithmic growth phase or at the beginning of the stationary phase (on the third day) and centrifuged at 4000× *g* for 20 min in an Avanti J-E centrifuge (Beckman Coulter, Bray, OK, USA). The supernatant was analyzed for the content of phytohormones (cytokinins, indole-3-acetic acid (IAA) and ABA). Three independent biological replicates were performed for each experiment.

Cytokinins from 2 mL of the supernatant of the bacterial liquid culture were twice extracted with n-butyl alcohol in a 2:1 ratio (aqueous phase/organic phase). The extract was evaporated to dryness. Cytokinin bases and their derivatives from the dry residue were separated by thin layer chromatography on silufol plates (Merck KGaA, Fluka, Darmstadt, Germany) in the system of solvents butanol: ammonium hydrate: water (6:1:2), according to [32]. In this work, we analyzed the riboside of zeatin (ZR, Rf 0.4–0.5) and zeatin (Z, Rf 0.6–0.7). The material from different zones was eluted and afterwards it was assayed by enzyme-linked immunosorbent assay (ELISA) using specific antibodies, as described earlier [32].

IAA and ABA from 1 mL of the supernatant of the bacterial culture liquid were extracted with diethyl ether, according to a modified scheme [33]. The IAA and ABA quantitative assay was performed with ELISA using specific antibodies, as described previously [34]. The reliability of the phytohormone immunoassay was confirmed using a dilution test and through a comparison with the data obtained with the results of high-performance liquid chromatography (HPLC) in combination with mass spectrometry [32,35].

### 2.5. Isolation of DNA and Identification of Lipopeptide Synthetase Genes in the B. subtilis Strains by PCR

Genomic DNA from bacteria was isolated with a lysis buffer containing 1% Chelex 100 resin (BioRad Laboratories, Hercules, CA, USA), 1% Triton X100, 1% Tween 20, and 0.005% cresol red. The genes of lipopeptide synthetase—phosphopantheteinyl transferase (*BsSfp*), surfactin synthetase (*BsSrf*), iturin synthetase (*BsItuA*, *BsItuB*) and fengycin synthetase (*BsFenD*)—were identified in bacterial strains and isolates using polymerase chain reaction (PCR) with gene–specific primers and in a TP4-PCR-01-”Tertsik” type amplifier (DNA Technology, Moscow, Russia). Primers to the *BsBac* gene encoding 16S RNA of *Bacillus* spp. were used as an internal control. PCR products were separated in 7% PAGE stained with ethidium bromide using GeneRuler DNA Ladder (Thermo Fisher Scientific, Waltham, MA, USA). The sequences of all the primers are presented in Table S1 (Supplementary Materials).

### 2.6. Isolation and Purification of the Lipopeptide-Rich Fraction (LRF) from the Liquid Culture Medium of B. subtilis Strains

Lipopeptide-rich fraction (LRF) from the liquid culture medium of bacteria was obtained using ethanol extraction [23,25]. After completion of cultivation, the bacterial suspension was centrifuged at 4000× *g* at 4 °C for 30 min in an Avanti J-E centrifuge (Beckman Coulter, Bray, OK, USA), the supernatant was acidified with by adding 2 M HCl to pH 2.0 and incubated overnight at 4 °C. The formed precipitate was washed with distilled water acidified to pH 2.0 with 2 M HCl and centrifuged twice at 4000× *g* for 30 min. The resulting precipitate was extracted twice with 80% ethanol (pH 7.0). The crude extract was purified using an Amicon Ultracel—3K filter (Merck KGaA, Darmstadt, Germany), the fraction with a molecular weight of less than 3 kDa was collected and dried on a vacuum concentrator (Eppendorf Concentrator 5301, Eppendorf, Hamburg, Germany) at 30 °C. The dried residue was weighed and subsequently re-dissolved in 80% ethanol and different concentrations were used in the experiments.

### 2.7. High-Performance Liquid Chromatography (HPLC) Analysis of the Bacillus Strains LRF

High-performance liquid chromatography of LRFs from the culture medium of Bs26D and Bs11VM was performed on the LC20-AT device (Shimadzu, Japan) equipped with the diode-matrix detector using the Discovery C18 column (25 cm × 4.6 mm × 5 microns). Chromatography was performed at column temperature 30 °C, the flow rate was 0.8 mL/min, and detection was carried out at a wavelength of 210 nm. When analyzing the content of surfactin, elution was performed with a mixture of water and 0.1% acetic acid in a ratio of 60:40 (eluent 1). For iturin, elution was carried out with a mixture of acetonitrile with 0.1% acetic acid in a ratio of 40:60 (eluent 2), as in [36]. Commercial surfactin (Surfactin from *B. subtilis*) and Iturin (Iturin A from *B. subtilis*) (Merck KGaA, Sigma-Aldrich, Darmstadt, Germany) were used as chromatographic standards.

### 2.8. Screening of Growth-Promoting Concentrations for the Bacillus Strains and Their LRFs

Growth-promoting concentrations for bacteria, LRFs and their compositions were assessed by seed germination, as well as by measuring fresh and dry biomass of three-day-old wheat seedlings. In each variant, 4 repetitions of 100 seeds were taken. As a control sample, pure, untreated with bacterial strains or LRFs seeds were used. Before treatment and sowing, the seeds were sterilized with a 10% hydrogen peroxide solution for 1 min. Then, before germination, the experimental wheat seeds were treated with a liquid culture of bacteria in a semi-dry manner. For this 1, 2, and 3 μL of an individual suspension with a titer of 2 × 10^9^ spores/mL per 1 g of seeds were diluted with 20 μL distilled water and the seeds were moistened with this solution, after which the seeds were left for several hours until completely dry. Thus, bacterial concentration were 2 × 10^6^ spores/mL (1), 4 × 10^6^ (2), 6 × 10^6^ (3). Lipopeptide-rich fractions (LRF) of Bs26D (LRF 26D) and Bs11VM (LRF 11VM) or solutions of their compositions (LRF 26D + LRF 11VM) at concentrations from 0.5 to 4.5 µg/mL were used to soak the seeds for 3 h. Seeds were germinated in Petri dishes on moistened filter paper in a thermostat at 24 °C. Evaluation and accounting of germinated seeds was carried out after 3 days. The results are presented as % germination of control.

In order to determine the fresh weight of plants, seedlings (10 seedlings without seed in each repetition) were slightly dried with filter paper and weighed. To determine the dry weight of plants, the seedlings were dried in a thermostat at a temperature of 80 °C to constant weight. The results are presented as fresh and dry weight of one seedling in mg, as well as in % of the control. The experimental data in tables were expressed as means ± SE, which were calculated in all treatments using MS Excel.

### 2.9. Experimental Design of Tripartite Bacteria-Aphid-Plant Interaction

Plant growth conditions: for each treatment option, the plants were grown in isolated plastic vessels by the hydroponic method on a 10% solution of Hoagland–Arnon nutrient medium in the KBW E6 plant growth chamber (Binder GmbH, Tuttlingen, Germany) at 20/24 °C (night/day) with illumination of 146 W/m^2^ PAR and 16 h photoperiod. To study biochemical characteristics of plants and transcriptional activity of genes, plants were grown in isolated 1-L plastic vessels with 50–70 plants in 400 mL of 10% Hoagland–Arnon solution on rafts wrapped in sterile filter paper. 4-days seedlings were populated with at least 10 aphids per plant. To prevent the migration of aphids, the vessels were closed with plastic insulators covered with a porous nonwoven material.

Seeds of control plants were soaked in distilled water (referred to as Control in tables, on graphs and histograms). Untreated control plants infested with aphids are referred to as “Water” in tables and figures. In each variant, 5 plants of populated and unpopulated with aphids were taken for research.

Bacterial treatment: To prove the effect of endophytic bacteria on plant defense and growth parameters, experimental wheat seeds were treated before sowing with a suspension culture of strains Bs26D, Bs11VM or their composition Bs26D + Bs11VM in a semi-dry manner in growth-promoting concentrations.

Lipopeptide-rich fractions treatment: Plant treatment with LRF was carried out to establish the role of LPs in the induction of protective signaling pathways in plants and did not pursue the goal of determining the duration of the effect of bacterial metabolites on the plant immune system. LRF 26D and LRF 11VM or their compositions (LRF 26D + LRF 11VM) were added to the nutrient medium of plants, so that the final concentration was growth—promoting, 24 h before the colonization of aphids. After 24 h, the medium was replaced with Hoagland–Arnon solution without LRFs.

### 2.10. Bioassay of Aphicidal Activity of the Bacillus Strains and Their LRF

Aphicidal activity of bacterial strains and their LRFs was tested using a method modified for wheat [37]. It was carried out on cut first leaves of wheat seedlings, placed in test tubes with 5 mL of the bacterial suspension at the concentration of 10^7^ spores/mL (control tubes contained 5 mL of sterile water) or with 5 mL of LRF at various concentrations from 1.5 to 150 µg/mL. Leaves were inhabited by aphids (10 wingless females per leaf). After 5 days, the number of dead and live aphids was counted. The aphicidal activity of the bacterial strains and LRF was expressed as mortality rate (%) among the total number of aphids.

### 2.11. Bioanalysis of the Different Types of Resistance to Aphids—Antibiosis and Endurance

Growth-promoting concentrations of bacterial suspensions and concentrations from 1.5 to 10 μg/mL of LRFs and their composition were used. To test the antibiosis, 5 plants were grown in separate isolated vessels for each treatment options (3 vessels per variant). Four-days-old wheat seedlings were populated with 1 aphid per plant. After 14 days, the absolute number of live aphids as well as the number of dead aphids was counted, [37]. The propagation coefficient (K) was calculated using the formula: K = average fecundity of the female during the experiment/duration of the experiment in days [37]. Fecundity and mortality were expressed as % of the total number of aphids.

To test the endurance, plants were grown individually in isolated vessels; there were 10 vessels for each treatment option. The length of four-days-old seedlings were measured from the level of the raft to the tip of the leaf, and then each plant was colonized with 20 wingless females and isolated. A constant number of aphids was maintained by removing excess aphids every 48 h for two weeks. At the end of the experiment after 14 days, the height of the first and second leaves of control plants and plants infested by aphids was measured; the results were compared with the initial measurement [37]. Endurance was expressed in % of leaf growth compared to unpopulated control.

### 2.12. Biochemical Parameters

To measure the hydrogen peroxide (H_2_O_2_) production and the activity of redox enzymes (peroxidase (POD) and catalase (CAT)), plant material (1:5 weight/volume) was fixed in liquid nitrogen 1 and 3 days after plant colonization by aphids. Plants were homogenized in 0.05 M solution of Na-phosphate buffer (PB), pH 6.2 and incubated at 4 °C for 30 min. Supernatants were separated by centrifugation at 15,000× *g* for 15 min (5415 K Eppendorf, Hamburg, Germany). Concentration of H_2_O_2_ in the supernatant was determined using xylenol orange in the presence of Fe^2+^ at 560 nm by the method [38]. POD activity was investigated in 96-well plates (Corning-Costar, Glendale, AZ, USA) by the oxidation of (o-) phenylenediamine in the presence of H_2_O_2_ at 490 nm on a Benchmark Microplate Reader spectrophotometer (Bio-Rad Laboratories, Hercules, CA, USA) [37]. The enzyme activity was expressed in optical density/mg of protein per minute. CAT activity was assessed based on the ability of H_2_O_2_ to form a stable-colored complex with molybdate salts [37]. Optical density was measured at 405 nm on a Benchmark Microplate Reader spectrophotometer. CAT activity was calculated using a calibration curve and expressed in µM H_2_O_2_/mg of protein per min. Protein content was determined by the Bradford method.

### 2.13. Isolation of RNA and Performing the Quantitative Real-Time Polymerase Chain Reaction (qPCR)

Leaves from five plants per biological replication were collected and fixed in liquid nitrogen 1 and 3 days after population with aphids. Total wheat RNA was extracted using TRIzol™ Reagent (Merck KGaA, Sigma-Aldrich, Darmstadt, Germany) according to the manufacturer’s instructions. cDNA synthesis was carried out as described previously [33]. Primers for qRT-PCR were designed using a web-based primer designing tool from IDT (http://eu.idtdna.com/Scitools/Applications/Primerquest, accessed on 10 November 2022) (USA). The sequences of all the primers are presented in Table S2 (Supplementary Materials). Quantitative PCR was performed by polymerase chain reaction in real time using a set of predefined reagents EvaGreenI (Synthol, Moscow, Russia) and CFX Connect real-time PCR Detection System device (BioRad Laboratories, Hercules, CA, USA). To standardize the data, wheat gene *TaRLI* (RNaseLinhibitor-like) (Table S2, Supplementary Materials) was used as an internal reference for the real-time qPCR analysis. The quantification of gene expression was performed using CFX Connect real-time PCR Detection System (BioRad Laboratories, Hercules, CA, USA). In order to quantify the relative gene expression using the delta-delta Ct method was performed as described earlier [33]. Three independent biological and three technical replications were performed for each experiment.

### 2.14. Statistical Analysis

All experiments were repeated 3 times with a different number of biological repetitions from 3 to 10. Experimental data were expressed as means ± SE, which were calculated in all treatments using MS Excel. The significance of differences was assessed by ANOVA followed by Duncan’s test (*p* ≤ 0.05) with STATISTICA 10.0 software.

## 3. Results

### 3.1. Characterization of B. subtilis 26D and B. subtilis 11VM Strains

#### 3.1.1. Production of Phytohormones by the Strain *B. subtilis* 11VM

The properties of Bs26D strain were described earlier [20,30,33]. The endophytic properties of strain Bs26D were tested on potato and tomato plants [29,33] and it was shown that the strain does not produce abscisic acid (ABA) in cultivation medium [33].

The Bs26D strain produced 0.15 µg/mL of cytokinins (the sum of zeatin and zeatin riboside), and 0.11 µg/mL of indoleacetic acid (IAA) in cultivation medium [33]. Our results showed that Bs11VM strain secreted two times more IAA and two times less cytokinins than Bs26D (Table 1). Previously, it was shown that Bs11VM had a high growth-promoting activity [39].

#### 3.1.2. Endophytic Rate and Antagonism of *B. subtilis* Strains to Each Other

Both bacteria were endophytic and were able to live in the internal tissues of wheat plants (Table 2). Bacteria Bs26D was found in wheat shoots in the amount of 1728.8 × 10^3^ CFU/g of fresh weight, which is two orders of magnitude higher than the value shown by Bs11VM (Table 2). The CFU numbers of Bs26D and Bs11VM in the wheat roots were broadly similar (297.7 and 424.7 × 10^3^ CFU/g of fresh weight) (Table 2).

In the case of the combined treatment of wheat plants with Bs26D and Bs11VM bacteria, the concentration of cells in the wheat shoots increased up to 3267.8 × 10^3^ CFU/g of fresh weight relative to their number during the individual treatment of plants, and mainly due to the Bs11VM (Table 2). The concentration of cells in the wheat roots was reduced relative to their number during individual plant treatment, but was of the same order (240.1 × 10^3^ CFU/g of fresh weight) (Table 2). Colonies isolated from wheat shoots after co-treatment with Bs26D + Bs11VM that grew in Petri dishes at the third dilution (10 colonies) were analyzed by RAPD analysis (Figure S1, Supplementary Materials). Using RAPD analysis, the ratio of strains Bs26D: Bs11VM in wheat shoots was 60%:40%, which amounted to in terms of CFU 1863.1 and 1413.4 × 10^3^ CFU/g of fresh weight, respectively (Figure S1, Supplemental Materials). Thus, during joint treatment, the Bs11VM strain increased the level of its endophytic rate by two orders of magnitude due to the presence of Bs26D strain.

To combine some bacterial strains into one biocontrol agent, the necessary property of each of them is the absence of antagonism between them. The antagonism of Bs26D and Bs11VM strains in relation to each other was studied by the method of perpendicular strokes. It was shown that Bs26D had a slight antagonistic effect on Bs11VM, and inhibited its growth on 3.5 mm distance near its own colonies (Table 3).

#### 3.1.3. Identification of Cyclic Lipopeptide Synthetases Genes of Endophytic Strains of *B. subtilis*

In strains Bs26D and Bs11VM, nonribosomal peptide synthetase (NRPS) genes encoding LP production were detected by PCR (Figure S2, Supplementary Materials). The analysis indicates that Bs26D contains NRPS gene clusters, which include *BsSfp*, *BsSrf1* genes involved in producing surfactin and Bs11VM contains genes, involved in producing iturin *BsItuA* and *BsItuB* (Figure S1, Supplementary Materials).

#### 3.1.4. Identification of the Cyclic Lipopeptides Produced by Endophytic Strains of *B. subtilis*

**Figure 1:**
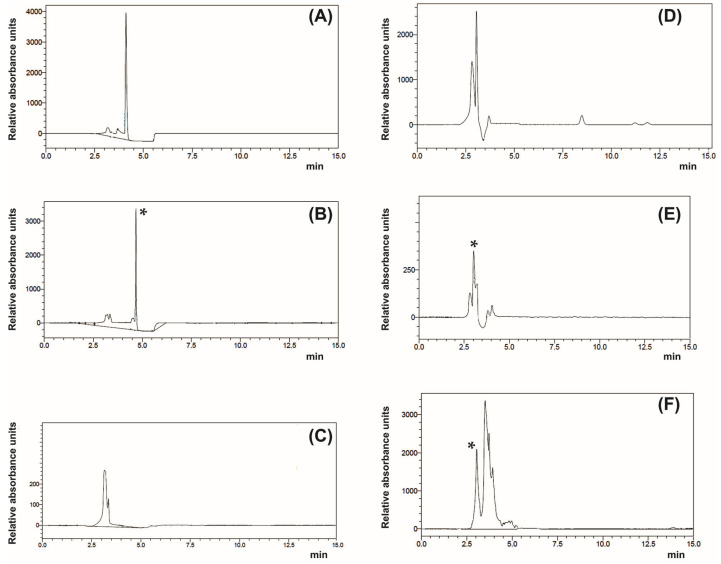
Chromatographic profiles of HPLC analysis of bacterial lipopeptide preparations (λ = 210 nm). (**A**)—commercial surfactin (Sigma-Aldrich, USA; 0.1 mg/mL); (**B**,**C**)—lipopeptide-containing fraction of the culture liquid *B. subtilis* 26D; (**D**)—commercial iturin (Sigma-Aldrich, United States; 0.1 mg/mL); (**E**,**F**)—lipopeptide-containing fraction of *B. subtilis* 11BM. An asterisk (*) marks the peaks corresponding to commercial surfactin (**B**) and iturin (**E**,**F**) in the lipopeptide samples.

High-performance liquid chromatography (HPLC) of lipopeptide-rich fraction (LRF) showed that cultural filtrate of Bs26D strain contained LP, which was identical to commercial surfactin (Figure 1A,B, Rt~4.5 min), and the cultural filtrate of Bs11VM strain contained LP, which was identical to commercial iturin (Figure 1D,F, Rt~2.5 min). Minor LPs were also found in cultural filtrate of Bs26D and Bs11VM strains (Figure 1C,E). Thus, surfactin and iturin are the main LPs of Bs26D and Bs11BM strains, respectively.

### 3.2. Screening of Wheat Growth-Promoting Concentrations of Suspensions of B. subtilis and Their LRFs

It is known that PGPB can actively influence plant growth. This fact is usually associated with the production of phytohormones by bacteria, as well as the ability to indirectly trigger a cascade of biochemical processes in plants, including accumulation of endogenous phytohormones [40]. In this regard, it became necessary to determine the growth-promoting concentrations of bacterial strains.

Seed treatment with bacterial strains Bs26D and Bs11VM increased seed germination by 10% and 6.5% compared to the control level, respectively (Table S3, Supplementary Materials). Moreover, the effect of bacterial strains on this indicator depended on the suspension concentration. Higher concentrations even inhibited seed germination. Interestingly, Bs11VM strain stimulated germination at lower concentrations (1 µL of bacterial suspension/g of seeds) than Bs26D strain (2 µL of bacterial suspension/g seeds) (Table S3, Supplementary Materials). The effect of LRF 26D and LRF 11VM on increasing seed germination was lower than that of bacterial suspensions (Table S4, Supplemental Materials).

Moreover, LRF 11VM worked at lower concentrations (1.5 μg/mL) than LRF 26D (2.5 μg/mL) (Table S4, Supplementary Materials). Increasing the concentration of LRF led to the inhibition of seed germination. Both Bs26D and Bs11VM bacterial strains and their LRF increased biomass of wheat seedlings depending on the concentration used, which coincided with the concentration, which promoted seed germination (Tables S3 and S4, Supplementary Materials). Thus, the growth-stimulating concentrations of bacterial strains and their LRFs were selected.

Subsequently, growth-promoting concentrations for the bacterial composition of Bs26D + Bs11VM and mixtures of their LRFs were selected (Table S5, Supplementary Materials). Since the effect of bacterial strains and their LRFs on seed germination and biomass accumulation depended on the concentration, in one case, the growth-promoting concentration for each bacterial strain or LRF was taken as the basis for compiling the compositions, and in the other case, these concentrations were reduced by 1.5–2 times (Table S5, Supplementary Materials). In most compositions, simply adding the growth-promoting concentrations of each component did not lead to promotion of seed germination. Reducing the growth-promoting concentration by 1.5 or 2 times led to stimulation of seed germination and better biomass accumulation (Table S5, Supplementary Materials). The effect of LRF 26D + LRF 11VM on the growth characteristics of wheat plants was investigated in three different combinations and concentrations (Table S5, Supplementary Materials). All three combinations had a positive effect on seed germination and accumulation of wheat biomass, however, the lowest concentrations of LRFs from both strains (2.0 + 1.5) µg/mL showed the best result (Table S5, Supplementary Materials). This combination of metabolites in composition was designated as growth-promoting concentration. In further work, selected growth-promoting concentrations of bacterial strains, their LRF and compositions were used (Table 4).

### 3.3. Aphicidal Activity of Endophytic Strains of B. subtilis and Their LRF

**Figure 2:**
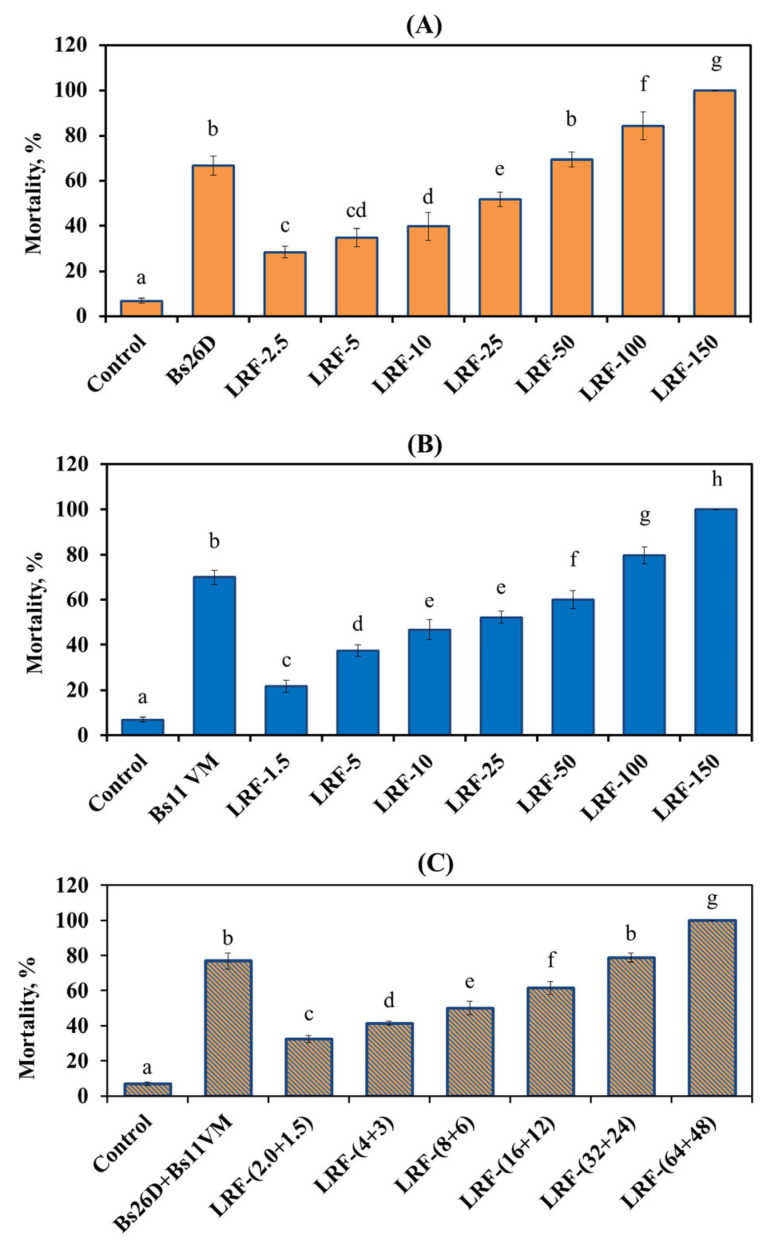
Aphicidal activity of strains of *B. subtilis* 26D (Bs26D) (**A**), *B. subtilis* 11VM (Bs11VM) (**B**), their composition (Bs26D + Bs11VM) (**C**) and the lipopeptide-rich fractions (LRFs) of the strains *B. subtilis* 26D (LRF 26D) (**A**), *B. subtilis* 11VM (LRF 11VM) (**B**) and the composition LRF 26D + LRF 11VM (**C**) against the greenbug aphid *S. graminum*. Concentrations used for individual LRFs 1.5, 2.5, 5, 10, 25, 50, 100 and 150 µg/mL, for LRFs mixture (2.0 + 1.5), (4 + 3), (8 + 6), (16 + 12), (32 + 24), (64 + 48) µg/mL. Figures present means ± SE (*n* = 5). Columns of each histogram marked with different letters represent the mean values that are statistically different from each other according to the Duncan’s test (*p ≤ 0.05*).

Bs26D and Bs11VM strains had rather high aphicidal activity: more than 65% of aphids did not survive when fed with a suspension of these strains for 5 days (Figure 2). The bacterial composition of Bs26D + Bs11VM showed an additive aphicidal effect (77% mortality rate) (Figure 2).

The insecticidal activity of bacterial strains against the greenbug aphid was manifested due to the synthesis of LPs by them. LRFs from both strains also had a negative effect on the viability of *S. graminum* when directly exposed (Figure 2). In this work, a direct relationship was found between the concentration of LRFs and the aphicidal effect of the studied LRFs. This dependence was observed within the concentration range from 2.5 to 150 µg/mL (Figure 2). The feeding of aphids on segments of wheat leaves immersed in solutions of LRF 26D or LRF 11VM (direct exposure) at low concentrations of 2.5–10 µg/mL caused from the death of 11 to 47% of aphids (Figure 2). A concentration of 25 μg/mL of LRF 26D or LRF 11VM caused the death of 50% of aphids, and 100% death of aphids was caused by 150 μg/mL already on the 5th day of feeding (Figure 2). Composition LRF 26D + LRF 11VM showed an additive aphicidal effect against *S. graminum*, especially at low concentrations (Figure 2). Thus, the growth-promoting concentration of the composition LRF 26D + LRF 11VM (2.0 + 1.5) µg/mL caused the death of 32.5% of aphids, the concentration (8 + 6) µg/mL caused the death of almost 50% of the pest, and 100% death of aphids was caused by 112 μg/mL (64 + 48) already on the 5th day of feeding (Figure 2).

### 3.4. Different Types of Defense against Aphids—Antibiosis and Endurance

In this work, the plant-mediated effect of Bs26D and Bs11VM strains and their LRFs on the viability of greenbug aphids was studied, which may be associated with the growth-promoting effect of these bacteria and with their metabolites, as well as their ability to induce immune reactions of plants [4,6,10]. This effect of bacteria can lead to an increase in plant endurance (tolerance) against aphids, which consists in the speed of restoration of photosynthetic activity and growth processes [5,41].

In the present experiments, the low endurance to *S. graminum* of wheat plants of the SY variety was established. Low endurance is manifested in the inhibition of the growth of the 1st and 2nd leaves to 82 and 70%, respectively, compared with the control non-infested with aphids plants (100%) (Table 5). The treatment of plants with a suspension of bacterial strains Bs26D, Bs11VM, or their composition, accelerated the growth of the 1st and 2nd leaves of wheat during aphid colonization (Table 5). In plants treated with bacteria, the presence of greenbug aphid did not inhibit growth; such plants grew even better than control plants by 3–42% (Table 5). It should be noted that the composition Bs26D + Bs11VM showed an additive effect on the growth of the 1^st^ leaf of wheat during the population of aphids (Table 5).

In addition, bacterial strains Bs26D, Bs11VM, or their composition, indirectly increased the mortality of aphids, reduced their fecundity and reproduction rate (propagation coefficient) when aphids fed on wheat plants treated with bacteria (Table 5). A significant decrease in the fecundity and reproduction rate of aphids on plants treated with the composition Bs26D + Bs11VM was a manifestation of an additive effect on these indicators (Table 5).

The indirect effect of different concentrations of LRF 26D, LRF 11VM, and the composition LRF 26D + LRF 11VM on plant endurance and aphid viability indicators was studied (Table 6). In this work, LRF concentrations of 1.5–10 µg/mL were studied. Growth-promoting concentrations of LRF 26D, LRF 11VM, and LRF 26D + LRF 11VM increased plant tolerance to the pest, but higher concentrations inhibited leaf growth of wheat colonized with greenbug aphid (Table 6). The strongest growth inhibition of the 1st leaf of wheat colonized with *S. graminum* was in plants treated with LRF 11VM at a concentration of 5 and 10 µg/mL (Table 6). At the same time, higher concentrations of LRF had a stronger effect on the viability of aphid (Table 6). Thus, aphid mortality increased from 24.9% to 38.3% in plants treated with LRF 26D at concentrations of 2.5 to 10 µg/mL, respectively (Table 6). In plants treated with LRF 11VM at concentrations from 1.5 to 10 µg/mL aphid fecundity decreased from 17.3 to 10.6 nymphs per plant, and mortality increased by 16.8% compared to water-treated plants (Table 6). However, the reproduction rate of aphids was already greatly reduced when exposed to low (growth-stimulating) LRF concentrations of both strains (Table 6).

Four different combinations of metabolite concentrations in the composition of LRF 26D + LRF 11VM affected the viability of aphids in a similar way (Table 6). The treatment option (2.5 + 2.5) µg/mL, where the content of LRFs from the culture medium of the strains was in an equal ratio (1: 1), mostly inhibited plant growth and had a lesser effect on the fecundity and reproduction rate of aphids than other combinations of LRFs (Table 6). An additive effect of LRF 26D + LRF 11VM composition on reproduction rate, aphid fecundity and 1st leaf growth was found in the case of the growth-promoting mixture (2.0 + 1.5) µg/mL concentration (Table 6).

### 3.5. The Effect of B. subtilis Strains, LRFs and Their Compositions on the Induction of Systemic Resistance in Wheat Plants Populated by Greenbug Aphid

#### 3.5.1. The Content of Hydrogen Peroxide and Activity of Redox Enzymes in Wheat Plants

The indirect effect of *B. subtilis* and their LRFs on plant endurance and viability indicators of aphids may be associated with the triggering of induced systemic resistance (ISR) in plants [5,37]. ISR is characterized by the early accumulation of reactive oxygen species (ROS) and changes in the redox status of plants, which leads to activation of gene expression and the development of defense reactions associated with the synthesis of defense proteins [5].

**Figure 3:**
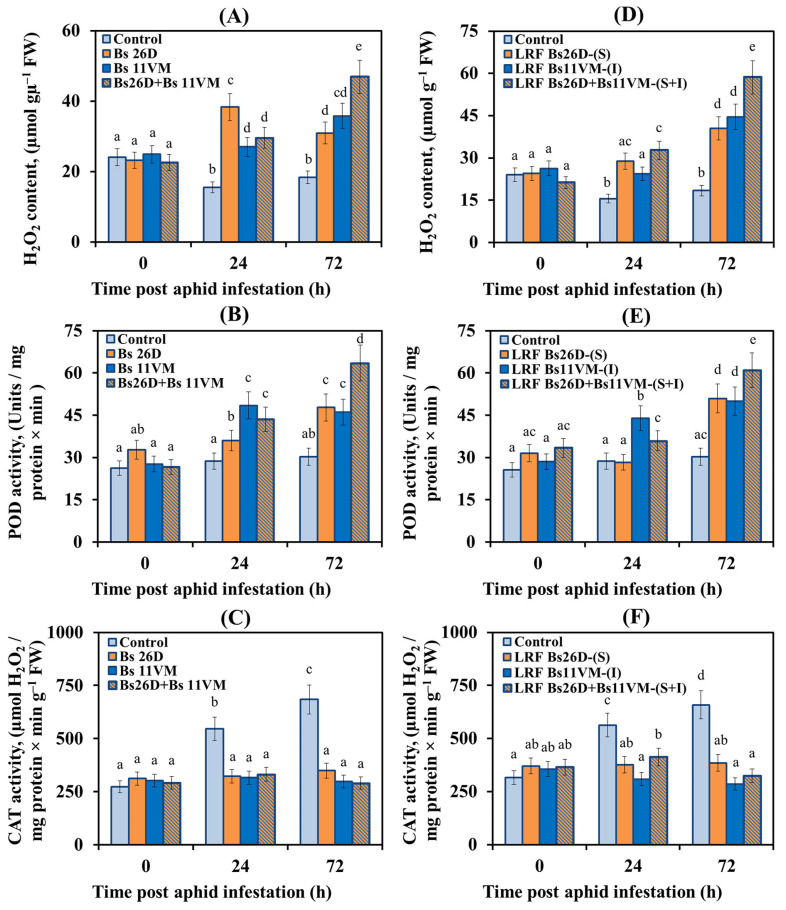
Influence of endophytic strains *B. subtilis* 26D (Bs 26D) and *B. subtilis* 11VM (Bs 11VM), lipopeptide-rich fraction (LRFs) and their compositions on the hydrogen peroxide (H_2_O_2_) content (**A**,**D**), peroxidase activity (POD) (**B**,**E**), and catalase activity (CAT) (**C**,**F**) of wheat plants infested with *S. graminum*. The samples are indicated as follows: 0 h—plants unpopulated with aphids; Control—unbacterized plants; Bs26D, Bs11VM and Bs26D + Bs11VM—plants treated with the appropriate strain or mixture of strains before sowing; LRF Bs26D-(S), LRF Bs11VM-(I), LRF Bs26D + Bs11VM-(S + I)—plants treated with the appropriate LRFs or their mixture 24 h before aphid infestation; (I)—iturin; (S)—surfactin. Figures present means ± SE (*n* = 6). Columns of each histogram marked with different letters represent the mean values that are statistically different from each other according to the Duncan’s test (*p* ≤ 0.05).

These results showed that H_2_O_2_ content decreased, peroxidase (POD) activity did not change, and catalase (CAT) activity significantly increased by more than two times in the initial stages of population with aphids of control wheat plants (Figure 3). In wheat plants treated with Bs26D and Bs11VM bacteria, or with a mixture of Bs26D + Bs11VM and infested with *S. graminum*, the H_2_O_2_ content and POD activity increased sharply, while CAT activity did not change compared to the control ones (Figure 3), which may have determined the resistance of such plants against the pest. The highest increase in the content of H_2_O_2_ was observed in plants treated with Bs26D cells 24 h post aphid infestation and in plants treated with the composition of strains 72 h post aphid infestation (Figure 3). The Bs11VM strain and the composition of Bs26D + Bs11VM strains had the greatest effect on the increase of POD activity 24 h post aphid infestation (Figure 3). Bacterial strains and their compositions had a similar effect on CAT activity (Figure 3).

The effect of LRFs on the components of the pro-/antioxidant system of wheat plants infested with greenbug aphid was similar to the effect of the strains themselves. It should be noted that LRF 11VM containing iturin (I) had the most significant effect on POD activity (Figure 3). An additive effect of the composition Bs26D + Bs11VM strains on the H_2_O_2_ content and POD activity in wheat plants populated with greenbug aphid was found (Figure 3).

#### 3.5.2. Expression of Redox Enzyme Genes

**Figure 4:**
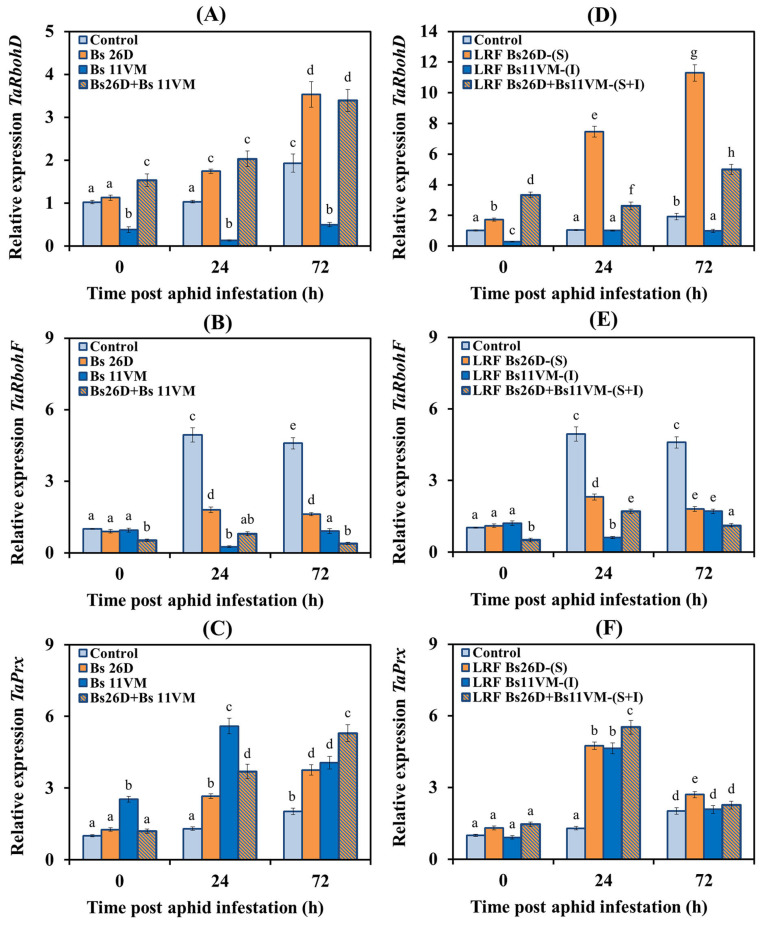
Influence of endophytic strains *B. subtilis* 26D (Bs26D) and *B. subtilis* 11VM (Bs11VM), lipopeptide-rich fraction (LRFs) and their compositions on the relative expression of genes *TaRbohD* (**A**,**D**), *TaRbohF* (**B**,**E**) and *TaPrx* (**C**,**F**) in wheat plants infested with *S. graminum*. The samples are indicated as follows: 0 h—plants unpopulated with aphids; Control—unbacterized plants; Bs26D, Bs11VM and Bs26D + Bs11VM—plants treated with the appropriate strain or mixture of strains before sowing; LRF Bs26D-(S), LRF Bs11VM-(I), LRF Bs26D+Bs11VM-(S+I)—plants treated with the appropriate LRFs or their mixture 24 h before aphid infestation; (I)—iturin; (S)—surfactin. Expression values were normalized to the housekeeping gene *TaRLI* as an internal reference and expressed relative to the normalized expression levels in control plants at 0 pai. Figures present means ± SE (*n* = 6). Columns of each histogram marked with different letters represent the mean values that are statistically different from each other according to the Duncan’s test (*p* ≤ 0.05).

The influence of Bs26D, Bs11VM strains, LRFs, and their compositions, on the transcriptional activity of *TaRbohD* and *TaRbohF* genes encoding NADPH oxidase isoforms, and the *TaPrx* gene encoding anionic peroxidase, has been studied. These results showed that the level of transcripts of the *TaRbohD* and *TaPrx* genes increased two times in non-bacterized wheat plants 72 h post aphid infestation compared to the control (Figure 4). At the same time, the content of mRNA of the *TaRbohF* gene increased five times in non-bacterized wheat plants populated with aphids (Figure 4).

The pre-sowing treatment of wheat seeds with the Bs26D strain led to a more significant accumulation of *TaRbohD* and *TaPrx* gene transcripts, and a less significant increase in the content of mRNA of *TaRbohF* gene than in water-treated plants colonized with aphids (Figure 4). The treatment of wheat seeds with Bs11VM led to a significant decrease in the transcript levels of *TaRbohD* and *TaRbohF* genes (Figure 4). However, 24 h post aphid infestation the mRNA content of the anionic peroxidase gene *TaPrx* increased by 5.6 times compared with the control in plants treated with Bs11VM, which was two times more than in plants treated with Bs26D and four times more than in water-treated colonized with aphids plants (Figure 4).

The treatment of wheat seeds with the composition of Bs26D + Bs11VM strains increased the expression of the *TaRbohD* and *TaPrx* genes just like Bs26D and decreased the expression of the *TaRbohF* gene just like Bs11VM individually (Figure 4). The effect of treatments of wheat plants with the bacterial strains Bs26D and Bs11VM on the expression of the *TaRbohD* and *TaRbohF* genes was opposite, and on the expression of the *TaPrx* gene, was similar (Figure 4). An additive effect of the composition of Bs26D + Bs11VM strains on *TaPrx* gene expression was found (Figure 4).

The effect of LRF 26D and LRF 11VM, which contained surfactin (S) and iturin (I), respectively, on the expression of *TaRbohD*, *TaRbohF,* and *TaPrx* genes, was similar to the effect of Bs26D and Bs11VM strains on this parameter, however, the degree of influence of LRFs and bacterial cells differed (Figure 4). Treatment with LRF 26D affected the accumulation of transcripts of the *TaRbohD* and *TaPrx* genes in plants populated by aphids much more strongly than treatment with Bs26D strain, the mRNA content increased by 11 and five times, respectively, compared to the control (Figure 4). The treatment of plants with LRF 11VM inhibited the accumulation of mRNA of *TaRbohF* gene and induced the accumulation of mRNA of *TaPrx* gene in plants populated with aphids (Figure 4). Plant treatment with LRF 26D + LRF 11VM increased the transcript level of the *TaRbohD* gene but to a lesser extent than Bs26D or Bs26D + Bs11VM, and affected the expression of *TaRbohF* and *TaPrx* genes similarly to LRF 26D and LRF 11VM (Figure 4).

#### 3.5.3. Expression of PR Proteins Genes Relating to Plant Hormone Signaling Pathways

**Figure 5:**
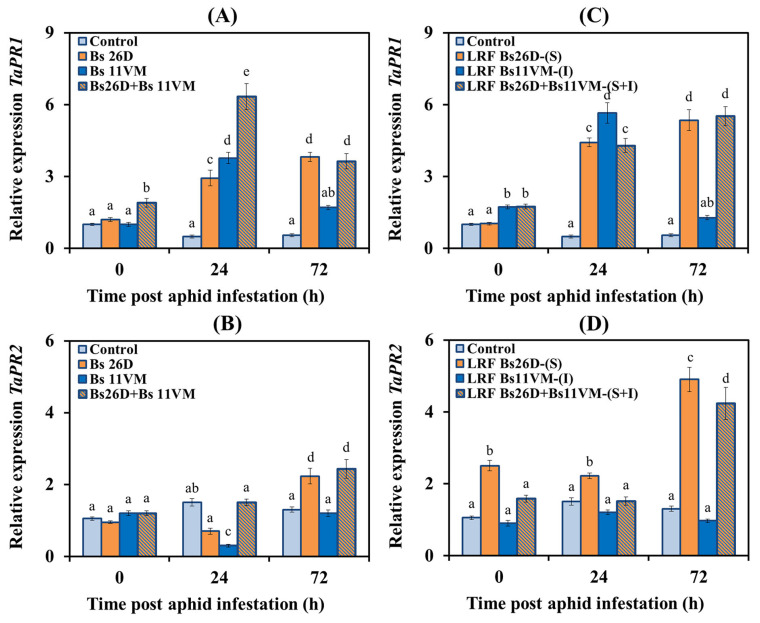
Influence of endophytic strains *B. subtilis* 26D (Bs26D) and *B. subtilis* 11VM (Bs11VM), lipopeptide-rich fraction (LRFs) and their compositions on the relative expression of SA-dependent genes *TaPR1* (**A**,**C**) and *TaPR2* (**B**,**D**) in wheat plants infested with *S. graminum*. The samples are indicated as follows: 0 h—plants unpopulated with aphids; Control—unbacterized plants; Bs26D, Bs11VM and Bs26D + Bs11VM—plants treated with the appropriate strain or mixture of strains before sowing; LRF Bs26D-(S), LRF Bs11VM-(I), LRF Bs26D + Bs11VM-(S + I)—plants treated with the appropriate LRFs or their mixture 24 h before aphid infestation; (I)—iturin; (S)—surfactin. Expression values were normalized to the housekeeping gene *TaRLI* as an internal reference and expressed relative to the normalized expression levels in control plants at 0 pai. Figures present means ± SE (*n* = 6). Columns of each histogram marked with different letters represent the mean values that are statistically different from each other according to the Duncan’s test (*p* ≤ 0.05).

**Figure 6:**
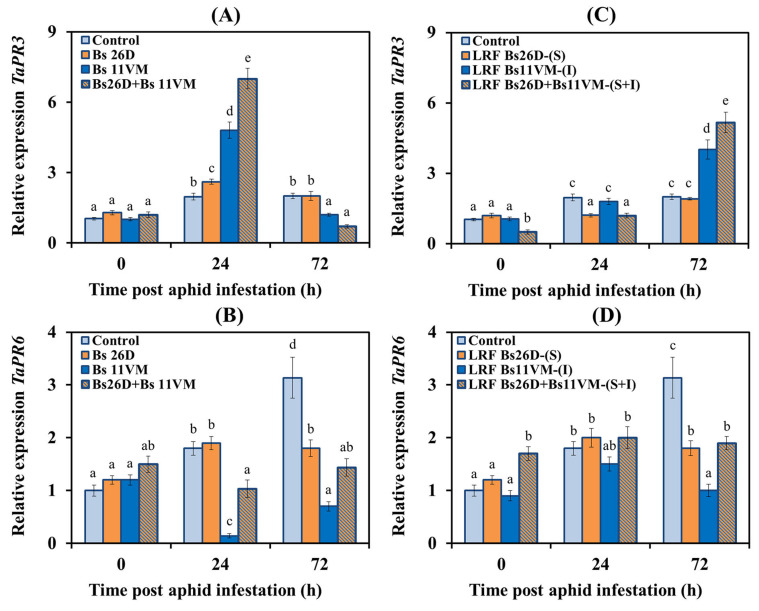
Influence of endophytic strains *B. subtilis* 26D (Bs26D) and *B. subtilis* 11VM (Bs11VM), lipopeptide-rich fraction (LRFs) and their compositions on the relative expression of ethylene-dependent gene *TaPR3* (**A**,**C**) and JA-dependent gene *TaPR6* (**B**,**D**) in wheat plants infested with *S. graminum*. The samples are indicated as follows: 0 h—plants unpopulated with aphids; Control—unbacterized plants; Bs26D, Bs11VM and Bs26D + Bs11VM—plants treated with the appropriate strain or mixture of strains before sowing; LRF Bs26D-(S), LRF Bs11VM-(I), LRF Bs26D + Bs11VM-(S + I)—plants treated with the appropriate LRFs or their mixture 24 h before aphid infestation; (I)—iturin; (S)—surfactin. Expression values were normalized to the housekeeping gene *TaRLI* as an internal reference and expressed relative to the normalized expression levels in control plants at 0 pai. Figures present means ± SE (*n* = 6). Columns of each histogram marked with different letters represent the mean values that are statistically different from each other according to the Duncan’s test (*p* ≤ 0.05).

In order to determine the ability of Bs26D, Bs11VM strains, LRFs, and their compositions, to regulate ISR in wheat plants against the greenbug aphid *S. graminum*, the expression of *PR1* and *PR2* genes, which are markers of the SA signaling pathway, *PR3* gene, which is the marker of the ethylene signaling pathway, and *PR6* gene, which is the marker of the JA signaling pathway, were studied [5,13]. Experimental results showed that the transcription of the *PR1* gene did not change, and the transcript level of the *PR2* gene increased insignificantly after the colonization of control plants with aphids, indicating that the SA signaling pathway was not activated (Figure 5). On the contrary, the JA/ethylene signaling pathway was activated already 24 h post aphid infestation—: mRNA content of the *PR3* and *PR6* genes increased 1.9 and 1.8 times, respectively, and an increase in the transcript levels of *PR6* gene by three times was found 72 h post aphid infestation compared to the control (Figure 6).

The pre-sowing treatment of wheat seeds with Bs26D strain led to a significant accumulation of mRNA of *TaPR1* and *TaPR2* genes by four and 2.2 times, respectively, in wheat plants populated with aphids compared to the control (Figure 5). Bs26D insignificantly affected the expression of *TaPR3* and *TaPR6* genes (Figure 6). The treatment of wheat seeds with the strain Bs11VM resulted in a significant increase in the mRNA content of *TaPR1* and *TaPR3* genes, by 3.8 and 4.8 times, respectively, and in a decrease in the transcripts level of the *TaPR2* and *TaPR6* genes (Figure 5 and Figure 6). The treatment of wheat seeds with the composition of strains Bs26D + Bs11VM increased the transcripts rate of *TaPR1*, *TaPR2,* and *TaPR3* genes (Figure 5 and Figure 6). The effect of treatments with bacterial strains Bs26D and Bs11VM on the expression of *TaPR2* and *TaPR3* genes was different, and on the expression of the *TaPR1* gene was similar (Figure 5 and Figure 6). An additive effect of the composition of Bs26D + Bs11VM strains on the expression of the *TaPR1* and *TaPR3* genes was found: the mRNA content increased by 6.3 and seven times, respectively (Figure 5 and Figure 6).

The effect of LRF 26D and LRF 11VM on *PR* genes expression was similar to the effect of Bs26D and Bs11VM strains on this parameter, however, the degree of influence of LRF and strains differed (Figure 5 and Figure 6). Treatment with LRF 26D affected the accumulation of levels of transcripts of the *TaPR1* and *TaPR2* genes in plants populated with aphids more than treatment with the Bs26D strain, the mRNA content increased five times for both genes (Figure 5). The treatment of plants with LRF 11VM induced mRNA accumulation of *TaPR1* gene by almost six times, which was much stronger than treatment with Bs11VM strain (Figure 5). The extent of induction of transcription of the *TaPR3* gene by LRF 11VM was the same as induction by the Bs11VM strain, but the expression induced by the metabolite was activated later (Figure 6). The treatment of plants with LRF 26D + LRF 11VM affected the expression of *PR* genes just like the composition of Bs26D + Bs11VM strains (Figure 5 and Figure 6). At the same time, the induction of the expression of the *TaPR2* gene by LRF 26D + LRF 11VM was stronger than after treatment with the composition of strains (Figure 5).

## 4. Discussion

The current work is focused on studying the mechanisms by which bacterial endophytes of *B. subtilis* individually and collectively protect plants from sucking insects, such as phloem-feeding aphids. Proposals have been put forward on the additive action of endophytes in the composition and the role of LPs in various processes has been considered.

The experimental results showed that the cells of Bs11VM strain did not penetrate well into the internal tissues of wheat and demonstrated an almost 29 times lower level of endophytic occurrence than the Bs26D strain (Table 2). However, under direct exposure, the aphicidity of Bs11VM against greenbug aphids was higher than that of Bs26D (Figure 2), while under indirect exposure, the effect of Bs11VM strain on aphid mortality and fertility was weaker than that of Bs26D (Table 5). The reason for this phenomenon may be related to the low ability of the Bs11VM strain to penetrate into the internal tissues of wheat. The additive aphicidal effect was observed upon direct influence of the composition of Bs26D + Bs11VM strains. (Figure 2); the composition also showed an additive effect when influencing the fertility and mortality of aphids with indirect exposure (Table 5). The mechanisms of endophyte penetration into host tissues have not been fully elucidated, but we assume that surfactin produced by Bs26D strain could contribute to an increase of the ability of bacterial cells of both strains to colonize the internal tissues of plants [42]. However, other mechanisms of this phenomenon cannot be excluded. Thus, recent reviews have described such metabolites as exopolysaccharides, hydrolytic enzymes, and others which are of great importance for the level of endophytic existence of bacteria [9,43].

According to the concept of direct and indirect immunity due to the microbiome, direct immunity is provided by the synthesis of bacterial metabolites [9,44]. Recently, *Bacillus* LPs have been considered as alternatives to synthetic chemical insecticides [18,19,44]. In the current work, surfactin and iturin isolated from cultural filtrate of endophytic bacteria Bs26D and Bs11VM, respectively (Figure 1), and showed approximately the same aphicidal activity against greenbug aphid *S. graminum* (Figure 2). Feeding aphids with solutions of LRF 26D (surfactin) or LRF 11VM (iturin) at a concentration of 150 μg/mL resulted in 100% mortality of the pest (Figure 2). Previously, the insecticidal activity of surfactin against several species of aphids (Hemiptera), cabbage moth *Plutella xylostella* (Lepidoptera), and cucumber beetle *Diabrotica balteata* (Coleoptera), was shown for the first time [45]. Later, surfactin was shown to be aphicidal against the green peach aphid *Myzus persicae* [18] and the rosy apple aphid *Dysaphis plantaginea* [21]. Recently, the insecticidal activity of other LPs, such as bacillopeptin and plipastatin (fengycin family), has been shown [21,22]. To date, the insecticidal and aphicidal activities of iturin have been shown only as part of the composition of LPs [19]. In this work, aphicidal activity was observed against bird cherry oat aphid *Rhopalosiphum padi* of a natural composition of LPs (surfactins, bacillomycins (iturin family), fengycin, iturin) isolated from the cultural filtrate of the bacterial strain *B. atrophaeus* L193 [19]. Moreover, in these works, lipopeptide-producing bacteria *Bacillus* spp. were not endophytes, with the exception of one study in which the endophytic strain *B. velezensis* YC7010 synthesizing bacillopeptin X conferred rice resistance against brown planthopper (*Nilaparvata lugens*) [22].

The composition of LPs (surfactin + iturin) was studied, which showed an additive aphicidal effect against *S. graminum* (Figure 2). There are only two works on the antibiotic activity of artificial compositions of LPs, one of which is devoted to the study of the fungicidal activity of fengycin + surfactin and mycosubtilin + surfactin composition against the pathogen the apple scab *Venturia inaequalis* [46], and the second focuses on the aphicidal activity of the triple composition plipastatin + mycosubtilin + surfactin (PMS) against rosy apple aphid *Dysaphis plantaginea* [21]. Thus, when studying the fungicidal activity of LPs, a composition of mycosubtilin + surfactin (80:20) showed a synergistic effect and good reproducibility of the results in field experiments against apple scab [46]. In the second study, the highest mortality of the rosy apple aphid was caused by surfactin, affecting the feeding behavior of the insect, while mycosubtilin and a composition of PMS in LP at ratio 33:33:33% caused the lowest mortality, but most of all affected the motoric activity of aphids [21].

Thus, screening of the concentrations and ratios between bacterial endophytes in compositions is a very important step in the selection of future bioinsecticides and important for understanding the mechanism of additive action. Various concentrations of bacterial strains and their LRF, were studied individually and in composition (Tables S3–5). The Bs11VM strain and LRF 11VM (iturin) showed growth-stimulating activity at lower concentrations than the Bs26D strain and LRF 26D (surfactin). In addition, with an increase in the concentration of strain or LPs, plant growth was inhibited (Tables S3 and S4). The best effect on the studied parameters was exerted by composition of strains Bs26D + Bs11VM in a ratio of 75:25% and a composition of LRF 26D + LRF 11VM in a ratio of 57:43% (Table S5, Table 5 and Table 6, Figure 2). A composition of LRF 26D + LRF 11VM proved to be the worst in a ratio of 50:50% (Table 6). In reviews of recent years, the mechanisms of plant growth stimulation by endophytes, including under biotic stress, are well described, which are associated with direct and indirect effects [47,48]. The direct effect is associated with the synthesis of phytohormones by bacteria and influence nutrient availability for plants, while the indirect effect is associated with the synthesis of antibiotic metabolites and the triggering of systemic resistance [47,48]. The regulation of the balance between the stimulation of plant growth and the induction of protective mechanisms in plants is the important issue about the mechanisms of the additive action of bacterial compositions.

Plant colonization by sucking insects that feed on phloem sap can cause plant growth inhibition and lead to severe yield losses of up to 80% [41]. The inhibition of plant growth when insects feed on phloem sap has been shown previously [41,49]. In the current work, the colonization of plants by *S. graminum* led to the inhibition of wheat leaf growth. The treatment of plants with endophytic strains Bs26D and Bs11VM in selected growth-stimulating concentrations increased the endurance (tolerance) of plants, accelerating leaf growth, while the composition Bs26D + Bs11VM showed an additive stimulating effect on plant growth (Table 4). Bacteria can influence plant growth by producing phytohormones, such as indole acetic acid, cytokinins and gibberellins [10,39,46,47]. Our results showed that the Bs26D strain secreted mainly cytokinins [32], while the Bs11VM strain secreted IAA into the culture medium (Table 1). It is possible that the composition Bs26D + Bs11VM showed an additive effect on plant growth due to the production of two phytohormones, cytokinins and IAA.

The indirect effect of endophytic strains on the growth of wheat plants could be associated with the synthesis of LPs and the triggering of systemic resistance [46,47]. In the present work, it has been shown, that bacterial LPs can be involved in increasing plant tolerance against the pest (Table 5). This can occur both due to the direct aphicidal action of LPs, which reduces the infectious load on plants, and due to the indirect effect associated with the induction of systemic resistance in plants.

The present work also shows that bacterial strains, LRFs, and their compositions, indirectly affected the vital parameters of greenbug aphids fed on treated wheat plants (Table 4 and Table 5). At the same time, the bacterial composition Bs26D + Bs11VM had an additive effect on the fecundity and reproduction rate of aphids (Table 4). Such an indirect effect of bacteria and their LRFs on aphid mortality may be associated with altering plant redox status and hormonal signaling and the triggering of induced systemic resistance (ISR). [5,9,14,22,23]. Thus, oxidative burst was considered as a typical reaction for the development of resistance to phloem-sap sucking insects [41].

An analysis of the state of the pro-/antioxidant system showed that the treatment of plants with bacterial strains, LRFs, and their compositions, caused an oxidative burst in plants populated by aphids (Figure 3)., The Bs26D strain and the compositions Bs26D + Bs11VM and LRF 26D + LRF 11VM had the greatest stimulatory effect on H_2_O_2_ accumulation (Figure 3). The protective role of H_2_O_2_ generation in response to aphids feeding on phloem sap may consist in the direct aphids’ injury by a high H_2_O_2_ level, and the indirect effect of H_2_O_2_ via the regulation of the ISR in damaged plants [5,22]. It has been reported that the exposure of *Arabidopsis* roots to the endophytic bacteria *B. velezensis* YC7010, could induce systemic resistance to aphids due to the increased H_2_O_2_ accumulation, cell death and deposition of callose in leaves [5].

In addition, the treatment of plants with bacterial strains, LRFs, and their compositions, increased POD activity and decreased CAT activity in aphid-infested plants (Figure 3). Bs11VM, LRF 11VM, and compositions Bs26D + Bs11VM and LRF 26D + LRF 11VM, had the greatest effect on POD activity (Figure 3). Previously, the activation of the apoplast peroxidases, in combination with a high H_2_O_2_ level, led to the reorganization and strengthening of the cellular walls due to lignification and the synthesis of phenols [41]. Low catalase activity in aphid-infected tolerant crop phenotypes [50], as well as downregulation of the transcriptional activity of the catalase gene in the resistant genotype of Arabidopsis infected with the green peach aphid *M. persicae* [51], facilitated the development of oxidative burst and tolerance.

Unfortunately, there are very few works describing the effect of bacteria, especially bacterial endophytes, on the activity of POD and other redox enzymes in insect-infested plants [22,37,52,53]. The cited works prove that bacteria-treated plants inoculated with insects exhibited increased POD activity, demonstrating the improved strategy for plant defense against insect induced by bacteria [22,37,52,53]. The effect of LPs on ROS generation and redox enzyme activity was described only during plant-fungal interaction [25,54,55]. These studies showed that surfactin, iturin, and fengycin, increased POD activity when plants were infected with various fungal pathogens [25,26,54], and surfactin + iturin most strongly induced POD activity [25].

In addition, the transcription activity of oxidoreductase genes involved in ROS generation *TaRbohD*, *TaRbohF* (membrane-bound NADPH oxidases) and *TaPrx* (apoplast-secreted peroxidase) has been studied (Figure 4). The role of NADPH oxidases in H_2_O_2_ generation in wheat plants populated by *S. graminum* has been shown recently [55,56]. The results of the present showed that the Bs26D, LRF 26D(S) and the compositions of Bs26D + Bs11VM and LRF 26D+ LRF 11VM increased the transcription activity of all *TaRbohD*, *TaRbohF* and *TaPrx* genes, which was accompanied by the highest level of H_2_O_2_ and oxidative burst in plants populated by aphids (Figure 3 and Figure 4). Bs26D cell suspension and LRF 26D (S) had the greatest effect on *TaRbohD* gene expression (Figure 4).

On the contrary, Bs11VM and LRF 11VM induced the expression of only the *TaPrx* gene, and the effect of LRF 11VM was transient, indicating the influence of other factors on the expression of this gene when plants were treated with Bs11VM (Figure 4). The expression of the *TaRbohD* and *TaRbohF* genes decreased in plants treated with Bs11VM and LRF 11VM (I) and colonized by greenbug aphids (Figure 4). It has been reported that surfactin and a surfactin + iturin mixture derived from *B. subtilis* ABS-S14 increased peroxidase expression in the fruits of *Citrus sinensis* inoculated with *Penicillium digitatum*, resulting in plant resistance to the disease [25]. Unfortunately, practically nothing is known about the effect of bacteria and their LPs on NADPH oxidase enzymes when plants are damaged by insects. It is known that the majority of ROS generated in response to avirulent *Pseudomonas syringae* bacteria and *Hyaloperonospora* sp oomycete pathogens depend on *RbohD* function, the induced cell death response induced by these pathogens appeared to be mostly regulated by *RbohF* [57]. Thus, the Bs26D + Bs11VM composition showed an additive effect in the induction of an oxidative burst, which could subsequently serve to enhance the ISR in wheat plants against aphids.

The analysis of transcriptional activity of genes-markers of SA, JA, and ethylene signaling pathways, showed that aphid feeding on control plants led to the activation of only genes related to the JA/ethylene-dependent response (*PR3* and *PR6*) (Figure 6). It has been shown that the first factor inducing the plant defense response to infection by sucking insect is mechanical injury, which requires the JA-dependent activation of lipoxygenase and proteinase inhibitors [58,59]. Previously, the activation of the JA signaling pathway has been observed in both aphid-susceptible and aphid-resistant plants, and the induction of the SA signaling pathway was faster and stronger in resistant genotypes [59]. The results of this study have shown that JA-dependent gene *PR6* was not activated in plants treated with bacterial suspensions or LRFs and infested with aphids (Figure 6).

The treatment of wheat with Bs26D and LRF 26D induced SA-dependent genes (*PR1* and *PR2*) in aphid-infested plants (Figure 5). The role of SA in the protective response to aphid feeding has been observed in many plant species [59]. Moreover, numerous studies have shown that the activation of the SA-signaling pathway may be a common antibiosis and aphid deterrent in resistant hosts [5]. We have previously shown that the induction of the SA pathway in wheat plants infested with *S. graminum* is important for development of resistance reactions [28].

Bs11VM cells and LRF 11VM (iturin) induced SA- and ethylene-dependent genes (*PR1* and *PR3*) (Figure 5 and Figure 6). Recently, it has been shown that during fungal pathogenesis, iturin induced SA-dependent genes *(PR1*, *PAL*) and ethylene-dependent genes (*PR3*, *AOC*, *ACS*), but not JA-dependent genes (*LOX*) [23,26]. Information on the role of ethylene in plant defense against aphids is much less and often contradictory. Some studies have observed an increase in ethylene levels in barley varieties which were resistant to *S. graminum* and *Rhopalophum padi* [60]. In another work, it was shown that ethylene signaling promoted aphid infestation on susceptible plants but contributed to antixenotic defenses that deterred the early stages of aphid-host interaction on resistant plants [61].

Treatments with Bs26D + Bs11VM showed an additive effect and induced the expression of *PR1* and *PR3* genes and, accordingly, induced SA and ethylene signaling pathways (Figure 5 and Figure 6), LRF 26D (surfactin) + LRF 11VM (iturin) showed an additive effect only in relation to *PR1* gene. Despite the increasing interest in bacteria-mediated ISR against leaf-chewing and phloem-feeding insects, the underlying molecular and chemical mechanisms of this phenomenon remain elusive [22,58,62,63]. It is known that different rhizobacteria genera including *Bacillus* and *Pseudomonas* have different effects on phloem-feeding insects. *B. subtilis* induced resistance against the phloem-feeding whitefly on tomato plants (*Solanum lycopersicum*), and increased the expression of both SA-dependent genes (PAL) and JA-dependent genes (LOX, IP) [62]. Rhizobacteria *P. simiae* WCS417r inhibited the reproduction of leaf-chewing insect *Mamestra brassicae* on *Arabidopsis thaliana* by triggering ISR giving preference to the JA/ET-regulated ORA59-branch, which led to the synthesis of camalexin and aliphatic glucosinolates (GLS) [58].

Thus, Bs26D and Bs11VM individually induced the transcriptional activity of PR genes of different signaling pathways in aphid-infested plants. The Bs26D + Bs11VM bacterial composition exhibited an additive effect in protecting plants against aphids, since it induced several hormonal signaling pathways, which is consistent with the assumptions of other authors [63]. In addition, the current study has shown for the first time that lipopeptides and their mixture induced the expression of defense genes in plants infested with greenbug aphid.

## 5. Conclusions

The present study showed that two endophytic lipopeptide- and hormone-producing strains of *B. subtilis* 26D and *B. subtilis* 11VM are able to induce the direct and indirect immunity of wheat plants against greenbug aphid *Schizaphis graminum*. *B. subtilis* 26D strain produces surfactin and cytokinins, and *B. subtilis* 11BM strain produces iturin and auxins. The development of direct defense mechanisms is ensured by the synthesis of lipopeptides and it is manifested in the high aphicidal activity of both strains. Treatment with endophytes induced ISR by activating the expression of markers of the SA- and ethylene-dependent *PR* genes, as well as due to the effect on the plant redox metabolism. This study shows for the first time the elicitor role of LPs surfactin and iturin in the induction of defense reactions in wheat against the aphid *S. graminum*. Additionally, in this study, it was shown for the first time that the composition of endophytic strains *B. subtilis* 26D + *B. subtilis* 11VM has additive effect on plant immunity due to the increase in the number of endophytic bacterial cells, and also due to the synergistic effect of the lipopeptides mixture − surfactin + iturin both on the aphid mortality and on the expression of *PR1* and *PR3* genes. All these factors can be the reason for the observed increase in the growth of plants affected by aphids under the influence of *B. subtilis* 26D and *B. subtilis* 11VM individually and in composition. It is worth noting that LPs of endophytic bacteria can play crucial roles in the development of direct and indirect bacterial-mediated mechanisms of plant defense. Further comprehensive investigations of the role of bacterial lipopeptides in endophyte–plant–aphid interactions will contribute to the development of new biotechnological, genetic, and breeding approaches to protect agricultural crops. Future research should use multi-omics approaches to isolate and identify more metabolites from endophytes, especially as plant resistance inducers, for increasing plant fitness and crop yields. The use of endophytes and their compositions to artificially develop stable plant microbiomes for plant protection has many advantages over chemical pesticides and traditional biocontrol agents, and is one of the most promising approaches for green pesticide discovery in the future.

## Figures and Tables

**Table 1:** Content of phytohormones in the culture medium of *B. subtilis* 11VM strain.

Strain	Phytohormone Level, µg/mL of Culture Medium		
	IAA	ABA	Cytokinins *
*B. subtilis* 11VM	0.31 ± 0.04	0.0	0.07 ± 0.006

* Cytokinins, the sum of zeatin and zeatin riboside. IAA—indoleacetic acid, ABA—abscisic acid.

**Table 2:** Content of microorganisms in the internal tissues of wheat seedlings.

Parameter	Part of Plant	Strain		
		*B. subtilis* 26D	*B. subtilis* 11VM	*B. subtilis* 26D + *B. subtilis* 11VM
Endophytic Rate, CFU × 10^3^/g of fresh weight	shoot	1728.8 ± 231.74 ^a^	60.3 ± 5.9 ^b^	3267.8 ± 316.09 ^c^
	root	297.7 ± 80.26 ^a^	424.7 ± 78.54 ^b^	240.1 ± 56.29 ^a^

CFU, colony-forming units. The variants in the same line marked with different letters represent the mean values that are statistically different from each other according to the Duncan’s test (*n* = 20, *p* ≤ 0.05).

**Table 3:** Antagonism of bacterial strains *B. subtilis* 26D and *B. subtilis* 11VM.

Strain	Distance from the Antagonist Colony, mm	
	Antagonist	
	*B. subtilis* 26D	*B. subtilis* 11VM
*B. subtilis* 26D	0	3.5 ± 3.6 ^a^
*B. subtilis* 11VM	3.5 ± 0.12 ^a^	0

The variants in the table marked with same letters represent the mean values that are not statistically different from each other according to the Duncan’s test (*n* = 5, *p* ≤ 0.05).

**Table 4:** Growth-promoting concentrations of suspensions of *B. subtilis* and their lipopeptide-rich fraction (LRF).

Strain or Strains Composition	Growth-Promoting Concentrations	
	Strain Concentration, µL/g Seeds	LRF Concentration, µg/mL
*B. subtilis* 26D	2.0	2.5
*B. subtilis* 11VM	1.0	1.5
*B. subtilis* 26D + *B. subtilis* 11VM	1.5 + 0.5	2.0 + 1.5

**Table 5:** Influence of endophytic strains *B. subtilis* 26D and *B. subtilis* 11VM and their compositions on the aphid viability indicators and endurance of wheat plants populated with *S. graminum*.

Variant of Treatment	Concentration of Bacterial Suspension, µl/g Seeds	Aphid Viability Indicators (Antibiosis)			Plants Endurance	
		Aphid Amount, (Nymphs/Seedling)	Mortality, %	Propagation Coefficient	Growth Rate of the 1st Leaf, % of Control *	Growth Rate of the 2nd Leaf, % of Control *
Water	-	36.8 ± 3.9 ^a^	6.9 ± 1.7 ^a^	2.45 ^a^	81.8 ± 6.2 ^a^	70.2 ± 5.1 ^a^
*B. subtilis* 26D	2.0	19.8 ± 2.2 ^b^	31.5 ± 2.2 ^b^	1.32 ^b^	114.7 ± 7.3 ^b^	142.0 ± 12.9 ^b^
*B. subtilis* 11VM	1.0	22.7 ± 2.8 ^b^	24.3 ± 3.4 ^c^	2.1 ^c^	103.2 ± 5.6 ^c^	115.0 ± 9.2 ^c^
*B. subtilis* 26D + *B. subtilis* 11VM	1.5 + 0.5	14.7 ± 1.9 ^c^	28.2 ± 2.6 ^b^	1.18 ^d^	120.5 ±6.8 ^d^	116 ± 5.3 ^c^

* Growth rate of the 1st or 2nd leaf of control, non-treated with bacterial suspensions and non-populated with aphids is 100%. The variants in the same column marked with different letters represent the mean values that are statistically different from each other according to the Duncan’s test (*n* = 15, *p* ≤ 0.05).

**Table 6:** Effect of lipopeptide-rich fraction (LRFs) and their compositions on the aphid viability indicators and endurance of wheat plants populated with *S. graminum*.

LRF from Strain and Their Mixture	Concentration of LRF, µg/mL	Aphid Viability Indicators (Antibiosis)			Plants Endurance	
		Aphid amount, (nymphs/Seedling)	Mortality, %	Propagation Coefficient	Growth Rate of the 1st Leaf, % of Control	Growth Rate of the 2nd Leaf, % of Control
Water	-	36.8 ± 3.9 ^a^	6.9 ± 1.7 ^a^	2.45 ^a^	81.8 ± 6.2 ^a^	70.2 ± 5.1 ^a^
LRF of *B. subtilis* 26D	2.5 *	20.0 ± 3.1 ^b^	24.9 ± 2.3 ^b^	1.3 ^b^	106.7 ± 5.7 ^b^	102.1 ± 4.8 ^b^
	5	19.4 ± 2.9 ^b^	33.7 ± 4.3 ^c^	1.26 ^b^	87.6 ± 5.8 ^c^	100.0 ± 4.1 ^b^
	10	15.6 ± 2.7 ^c^	37.3 ± 4.8 ^d^	1.26 ^b^	86.5 ± 6.1 ^c^	94.7 ± 3.2 ^c^
LRF of *B. subtilis* 11VM	1.5 *	17.3 ± 3.3 ^bc^	20.9 ± 2.6 ^b^	1.2 ^b^	98.1 ± 6.2 ^d^	102.3 ± 7.6 ^b^
	2.5	16.3 ± 4.1 ^c^	29.3 ± 2.7 ^c^	1.3 ^b^	96.9 ± 6.3 ^d^	101.8 ± 4.5 ^b^
	3.5	16.8 ± 1.8 ^c^	30.5 ± 4.1 ^c^	1.06 ^c^	91.2 ± 6.0 ^c^	90.8 ± 4.6 ^c^
	5	14.1 ± 3.3 ^c^	34.9 ± 3.9 ^c^	1.09 ^c^	75.0 ± 4.4 ^e^	72.3 ± 3.3 ^d^
	10	10.6 ± 3.2 ^d^	39.7 ± 5.1 ^d^	1.07 ^c^	73.4 ± 4.2 ^e^	71.5 ± 4.1 ^d^
LRF of *B. subtilis* 26D + *B. subtilis* 11VM	2.0 + 1.5 *	16.3 ± 1.2 ^c^	26.3 ± 3.1 ^b^	1.06 ^c^	110.2 ± 5.1 ^b^	100.3 ± 3.8 ^b^
	2.5 + 1.5	16.5 ± 2.8 ^c^	34.6 ± 3.5 ^c^	1.07 ^c^	95.0 ± 5.8 ^d^	103.9 ± 4.8 ^b^
	2.5 + 2.5	20.6 ± 3.5 ^b^	34.2 ± 3.7 ^c^	1.6 ^d^	90.8 ± 5.6 ^c^	93.8 ± 4.2 ^c^
	2.5 + 3.5	16.4 ± 1.9 ^c^	31.5 ± 4.1 ^c^	1.06 ^c^	94.1 ± 6.1 ^cd^	94.8 ± 4.5 ^c^

* Growth-promoting concentrations of LRF. The variants in the same column marked with different letters represent the mean values that are statistically different from each other according to the Duncan’s test (*n* = 15, *p ≤ 0.05*).

## Data Availability

Not applicable.

## Supplementary Materials

The following supporting information can be downloaded at: https://www.mdpi.com/article/10.3390/life13010214/s1, Table S1: Primers used for PCR.; Table S2: Primers used for qPCR; Figure S1: RAPD analysis of DNA of bacterial colonies from plants treated with a mixture of *B. subtilis* 26D + *B. subtilis* 11VM: (A) DNA of the original bacterial strain *B. subtilis* 11VM; (B)—DNA of the original bacterial strain *B. subtilis* 26D; 1–10—DNA of bacterial strains isolated from ten colonies that grew in a Petri dish in the third dilution, from an aliquot of sterile plant homogenate (1–4, 7, 9—*B. subtilis* 26D; 5, 6, 8, 10—*B. subtilis* 11VM). The red asterix (*) represents strain *B. subtilis* 26B, blue asterix (*) indicates strain *B. subtilis* 11VM; Figure S2: Screening of strains *B. subtilis* 26D (A) and *B. subtilis* 11VM (B) for the presence of genes encoding lipopeptide synthase: *Srf*—phosphopantheteinyl transferase; *Sfp*—surfactin synthase; *ItuA* and *ItuB*—iturin synthase; *Fen*D—fengycin synthase; *Bac*—reference gene; Table S3: Influence of endophytic strains *B. subtilis* 26D and *B. subtilis* 11VM on the seed germination and biomass accumulation; Table S4: Effect of lipopeptide-rich fractions (LRFs) on the seed germination and biomass accumulation; Table S5: Influence of compositions endophytic strains *B. subtilis* 26D and *B. subtilis* 11VM and their lipopeptide-rich fractions (LRFs) on the seed germination and biomass accumulation.
