# Supplementary material for: Additive Effect of the Composition of Endophytic Bacteria Bacillus subtilis on Systemic Resistance of Wheat against Greenbug Aphid Schizaphis graminum Due to Lipopeptides
Source: Life (Basel). 2023 Jan 11;13(1):214. doi: 10.3390/life13010214 (PMC9860984; doi:10.3390/life13010214)
Supplement: Supplementary file 1 [file life-13-00214-s001.zip › life-2067847-supplementary.pdf]

Additive Effect of the Composition of Endophytic Bacteria *Bacillus subtilis* on Systemic Resistance of Wheat against Greenbug Aphid *Schizaphis graminum* Due to Lipopeptides

Sergey D. Rumyantsev, Valentin Y. Alekseev, Antonina V. Sorokan, Guzel F. Burkhanova, Ekaterina A. Cherepanova, Ravil R. Garafutdinov, Igor V. Maksimov and Svetlana V. Veselova*

Institute of Biochemistry and Genetics, Ufa Federal Research Centre, Russian Academy of Sciences, Prospekt Oktyabrya, 71, 450054 Ufa, Russia

* Correspondence: veselova75@rambler.ru; Tel.; +7(917)3423941

Supplementary Table S1. Primers used for PCR

Gene's product	Genes	GenBank Accession number	Sequence (5'-3')	
			**Forward Primers**	**Reverse Primer**
phosphopantheteinyl transferase	*Bs_sfp*	KT750873	ATGAAGATTTACGGAATTTA	TTATAAAAGCTCTTCGTACG
surfactin synthetase	*Bs_srf*	EU882341	ATGAAGATTTACGGAATTTATATG	TTATAAAAGCTCTTCGTACGAG
iturin synthetase A	*Bs_ituA*	D21876	ATGAAAATTTACGGAGTATATATG	TTATAACAGCTCTTCATACGTT
iturin synthetase B	*Bs_ituB*	KR149331	AAGAAGGCGTTTTTCAAGCA	CGACATACAGTTCTCCCGGT
fengycin synthetase	*Bs_fen*	AJ011849	TTTGGCAGCAGGAGAAGTTT	GCTGTCCGTTCTGCTTTTTC
16S ribosomal RNA	*Bs_Bac*	NR102783	ACCAGAAAGCCACGGCTAACTAC	GGCGGAAACCCCCTAACACT

Supplementary Table S2. Primers used for qPCR

Gene's product	Genes	GenBank Accession number	Sequence (5'-3')	
			**Forward Primers**	**Reverse Primer**
RNase L inhibitor protein	*TaRLI*	AY059462	TTGAGCAACTCATGGACCAG	GCTTTCCAAGGCACAAACAT
Lipoxygenase	*TaLOX*	BJ223744	AGTCCGAGAAAACATGGCGTC	ATAGTCCGAGACTCCAAG
Phenylalanine ammonia-lyase	*TaPAL*	X99725	GGCGTCAAAACATGGCGTC	AGTCCGAGAAGTCCGAGA
PR1	*T PR1*	AF384143	ATAACCTCGGCGTCTTCATC	GCTTATTACGGCATTCCTTTT
PR2	*TaPR2*	DQ090946	GCGTGAAGGTGGTGATTT	GTGCCCGTTACACTTGGAT
PR3	*TaPR3*	AB029936	ACCTCCTTGGCGTCAGCT	TCGCACCATTATTCCCTT
Proteinase inhibitor, PR6	*TaPR6*	EU293132	GGGCCCTGCAAGAAGTACTG	ACACGCATAGGCACGATGAC
Peroxidase, PR9	*TaPR9*	TC 151917	TCGACAAGCAGTACTACCACAA	CCGAAGTCCGAGAAGAACTG
NADPH oxidase F	*TaRbohF*	AY561153	ATGTTCGGCAACTTGGTGACT	CGTCTGCTCTAAGAAGACCACTTTT
NADPH oxidase D	*TaRbohD*	AK335454	ACCACCAGACCAGACCAGAC	TGGTTGGATAGGAGGCGTAG

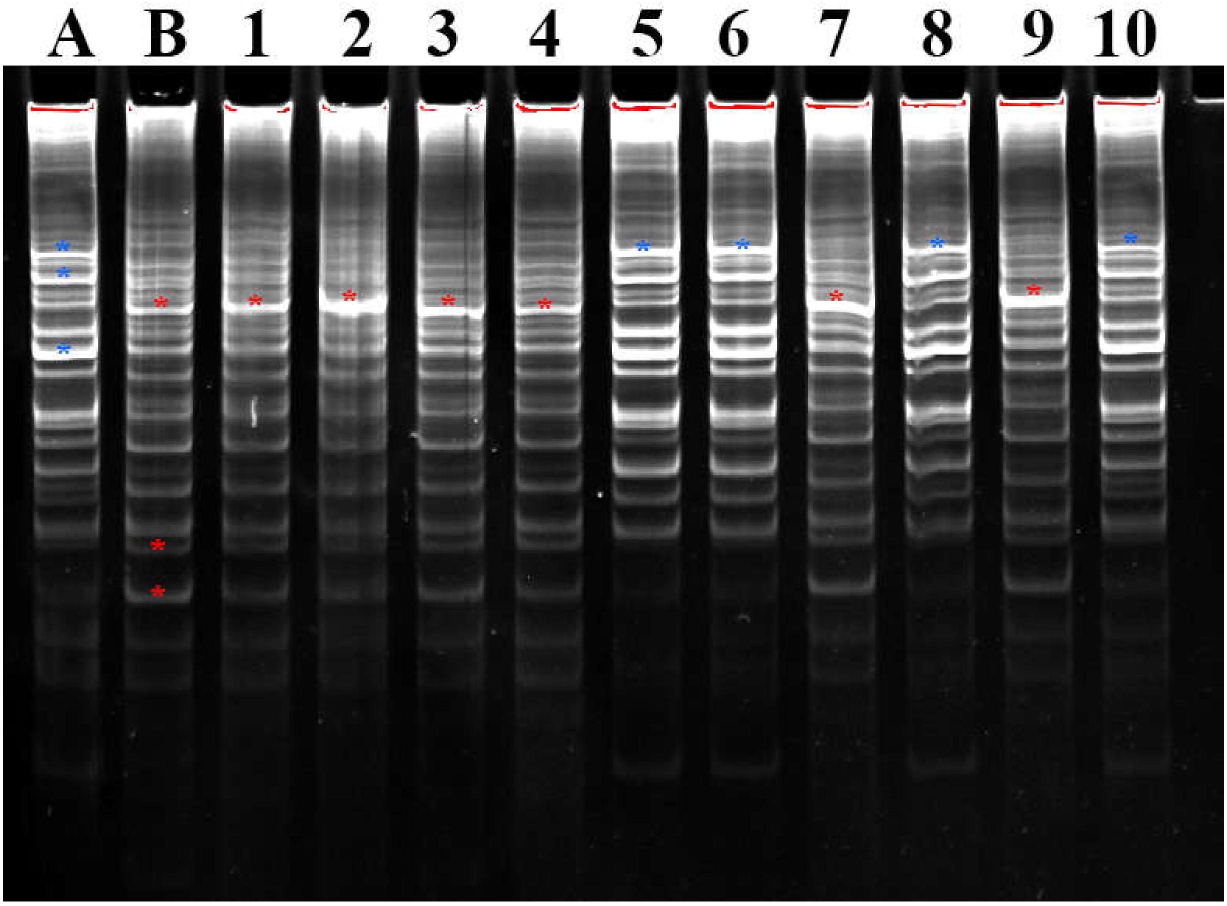

Supplementary Figure S1. RAPD analysis of DNA of bacterial colonies from plants treated with a mixture of *B. subtilis* 26D + *B. subtilis* 11VM: (**A**) DNA of the original bacterial strain *B. subtilis* 11VM; (**B**) – DNA of the original bacterial strain *B. subtilis* 26D; 1-10 - DNA of bacterial strains isolated from ten colonies that grew in a Petri dish in the third dilution, from an aliquot of sterile plant homogenate (1-4, 7, 9 - *B. subtilis* 26D; 5, 6, 8, 10 - *B .subtilis* 11VM). The red asterix (*) represents strain *B .subtilis* 26B, blue asterix (*) indicates strain *B .subtilis* 11VM.

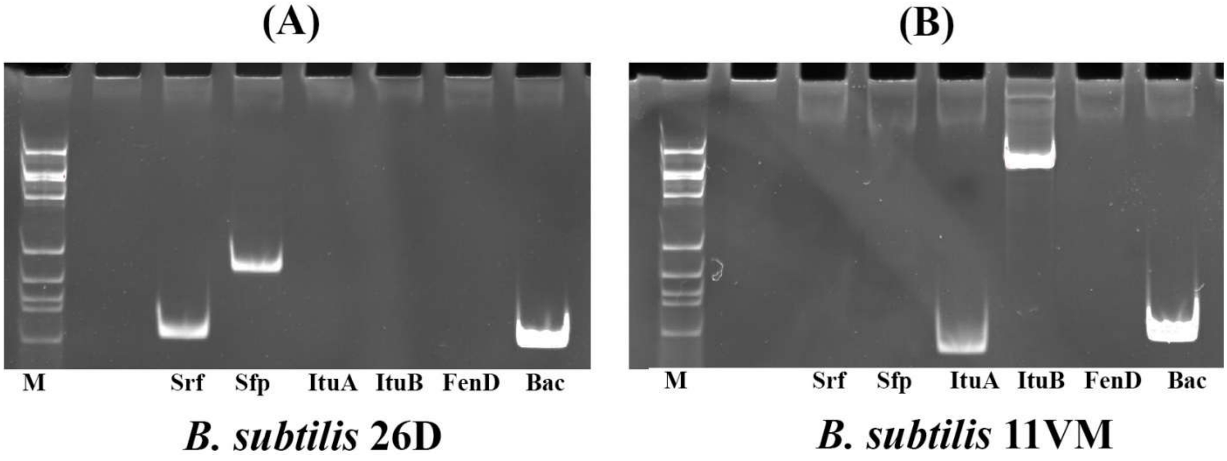

Supplementary Figure S2. Screening of strains *B. subtilis* 26D (A) and *B. subtilis* 11VM (B) for the presence of genes encoding lipopeptide synthase: *Srf* - phosphopantheteinyl transferase; *Sfp* - surfactin synthase; *ItuA* and *ItuB* - iturin synthase; *Fen*D - fengycin synthase; Bac - reference gene.

Supplementary Table S3. Influence of endophytic strains *B. subtilis* 26D and *B. subtilis* 11VM on the seed germination and biomass accumulation.

Parameter	Variant of treatment			
	**Control**	**Concentration of bacterial suspension, μl/g seeds**	***B. subtilis* 26D**	***B. subtilis* 11VM**
**Germination, %**	90.0 ± 2.8 [a]	1	90.1 ± 2.9 [a]	96.5 ± 2.1 [c]
		2	99.6 ± 2.5 [b]	93.7 ± 3.4 [d]
		3	92.2 ± 3.1 [a]	90.6 ± 3.6 [a]
**Fresh weight of one seedling, mg**	62.5 ± 7.2 [a]	1	66.7 ± 1.5 [b]	72.5 ± 1.1 [d]
		2	80.2 ± 3.2 [c]	62.1 ± 0.5 [a]
		3	71.8 ± 7.7 [d]	66.5 ± 0.3 [b]
**Increment of fresh weight of one seedling, % of control**	100 ± 10.0 [a]	1	107.0 ± 1.2 [b]	116.0 ± 2.1 [d]
		2	129.0 ± 4.6 [c]	100.0 ± 1.4 [a]
		3	115.0 ± 6.0 [d]	107.0 ± 1.1 [b]
**Dry weight of one seedling, mg**	5.4 ± 0.5 [a]	1	6.3 ± 0.1 [b]	6.8 ± 0.1 [c]
		2	7.1 ± 0.1 [c]	6.0 ± 0.2 [b]
		3	7.1 ± 0.1 [c]	6.2 ± 0.2 [b]
**Increment of dry weight of one seedling, % of control**	100 ± 9.0 [a]	1	116.0 ± 2.3 [b]	126.0 ± 1.4 [c]
		2	132.0 ± 1.8 [c]	110.0 ± 3.1 [b]
		3	131.0 ± 1.9 [c]	115.0 ± 4.2 [b]

The variants in the same column marked with different letters represent the mean values that are statistically different from each other according to the Duncan's test ($n = 40$, $p \leq 0.05$).

Supplementary Table S4. Effect of lipopeptide-rich fractions (LRFs) on the seed germination and biomass accumulation.

Parameter	Variant of treatment			
	Control	Concentration of lipopeptide-rich fraction, μg/mL	LRF of *B. subtilis* 26D	LRF of *B. subtilis* 11VM
**Germination, %**	90.0 ± 2.8 [a]	0.5	91.1 ± 2.5 [a]	93.5 ± 2.9 [c]
		1.5	91.5 ± 2.2 [a]	95.9 ± 3.3 [c]
		2.5	96.1 ± 3.1 [b]	92.2 ± 2.4 [a]
		3.5	92.5 ± 2.7 [a]	90.3 ± 1.8 [a]
		4.5	90.7 ± 2.1 [a]	89.9 ± 2.0 [a]
**Fresh weight of one seedling, mg**	64.3 ± 10.8 [a]	0.5	67.8 ± 3.7 [a]	98.2 ± 0.8 [e]
		1.5	76.6 ± 1.5 [b]	102.0 ± 2.9 [e]
		2.5	87.5 ± 6.9 [c]	81.5 ± 0.9 [c]
		3.5	64.1 ± 2.5 [a]	81.2 ± 1.6 [c]
		4.5	54.9 ± 1.8 [d]	77.2 ± 1.2 [b]
**Increment of fresh weight of one seedling. % of control**	100 ± 12.0 [a]	0.5	105.0 ± 3.1 [a]	153.0 ± 1.5 [e]
		1.5	119.0 ± 2.0 [b]	159.0 ± 5.1 [e]
		2.5	136.0 ± 5.0 [c]	127.0 ± 2.0 [c]
		3.5	100.0 ± 4.1 [a]	126.0 ± 2.1 [c]
		4.5	85.0 ± 3.2 [d]	120.0 ± 2.0 [b]
**Dry weight of one seedling. mg**	5.8 ± 0.6 [a]	0.5	6.3 ± 0.1 [b]	7.7 ± 0.1 [d]
		1.5	7.0 ± 0.2 [c]	8.2 ± 0.1 [e]
		2.5	7.4 ± 0.3 [c]	7.1 ± 0.3 [d]
		3.5	6.2 ± 0.1 [b]	7.1 ± 0.1 [c]
		4.5	5.7 ± 0.1 [a]	6.3 ± 0.1 [b]
**Increment of dry weight of one seedling. % of control**	100 ± 10.0 [a]	0.5	109.0 ± 1.2 [b]	132.0 ± 1.0 [d]
		1.5	121.0 ± 4.0 [c]	142.0 ± 2.0 [e]
		2.5	127.0 ± 6.0 [c]	122.0 ± 4.5 [d]
		3.5	106.0 ± 1.5 [b]	122.0 ± 2.2 [c]
		4.5	98.0 ± 2.0 [a]	109.0 ± 2.0 [b]

The variants in the same column marked with different letters represent the mean values that are statistically different from each other according to the Duncan's test ($n = 40$, $p \leq 0.05$).

Supplementary Table S5. Influence of compositions endophytic strains *B. subtilis* 26D and *B. subtilis* 11VM and their lipopeptide-rich fractions (LRFs) on the seed germination and biomass accumulation.

Parameter	Variant of treatment				
	Control	Concentration of bacterial suspension, µl/g seeds	*B. subtilis* 26D + *B. subtilis* 11VM	Concentration of lipopeptide-rich fraction, µg/mL	LRF of *B. subtilis* 26D + *B. subtilis* 11VM
**Germination, %**	90.0 ± 2.8 [a]	1.5 + 0.5	99.6 ± 2.5[b]	2.0 + 1.5	96.5 ± 2.1 [b]
		1.5 + 1.0	92.2 ± 3.1[a]	2.5 + 1.5	93.7 ± 3.4 [c]
		2.0 + 1.0	90.1 ± 2.9[a]	2.5 + 3.5	90.6 ± 3.6 [a]
**Fresh weight of one seedling, mg**	62.5 ± 7.2 [a]	1.5 + 0.5	80.2 ± 3.2 [b]	2.0 + 1.5	72.5 ± 1.1 [b]
		1.5 + 1.0	71.8 ± 7.7 [c]	2.5 + 1.5	68.5 ± 0.3 [b]
		2.0 + 1.0	66.7 ± 1.5 [d]	2.5 + 3.5	62.1 ± 0.5 [a]
**Increment of fresh weight of one seedling, % of control**	100 ± 10.0 [a]	1.5 + 0.5	129.0 ± 4.6 [b]	2.0 + 1.5	116.0 ± 2.1 [b]
		1.5 + 1.0	115.0 ± 6.0 [c]	2.5 + 1.5	109.6 ± 1.1 [b]
		2.0 + 1.0	107.0 ± 1.2 [d]	2.5 + 3.5	99.4 ± 1.4 [a]
**Dry weight of one seedling, mg**	5.4 ± 0.5 [a]	1.5 + 0.5	7.1 ± 0.1 [b]	2.0 + 1.5	6.8 ± 0.1 [b]
		1.5 + 1.0	6.2 ± 0.1 [c]	2.5 + 1.5	6.2 ± 0.2 [c]
		2.0 + 1.0	6.3 ± 0.1 [c]	2.5 + 3.5	6.0 ± 0.2 [c]
**Increment of dry weight of one seedling, % of control**	100 ± 9.0 [a]	1.5 + 0.5	132.0 ± 1.8 [b]	2.0 + 1.5	126.0 ± 1.4 [b]
		1.5 + 1.0	114.8 ± 1.9 [c]	2.5 + 1.5	114.8 ± 4.2 [c]
		2.0 + 1.0	116.0 ± 2.3 [c]	2.5 + 3.5	111.0 ± 3.1 [c]

The variants in the same column marked with different letters represent the mean values that are statistically different from each other according to the Duncan’s test ($n = 40$, $p \leq 0.05$).
